# Comparative Analysis of Within-Host Dynamics of Acute Infection and Viral Rebound Dynamics in Postnatally SHIV-Infected ART-Treated Infant Rhesus Macaques

**DOI:** 10.3390/v18101076

**Published:** 2026-09-29

**Authors:** Ellie Mainou, Stella J. Berendam, Veronica Obregon-Perko, Emilie A. Russell, Caroline T. Phan, George M. Shaw, Katharine J. Bar, Mithra R. Kumar, Emily J. Fray, Janet M. Siliciano, Robert F. Siliciano, Guido Silvestri, Sallie R. Permar, Genevieve G. Fouda, Janice M. McCarthy, Ann Chahroudi, Cliburn Chan, Jessica M. Conway

**Affiliations:** 1 Department of Biology, Pennsylvania State University, University Park, PA 16801, USA; 2 GlaxoSmithKline, Rockville, MD 20850, USA; berendamstellaj@gmail.com; 3 FlowJo, Atlanta, GA 30322, USA; veronica.obregon-perko@bd.com; 4 Duke Human Vaccine Institute, Duke University Medical Center, Durham, NC 27710, USA; emilieuffman@gmail.com (E.A.R.); carolph@uw.edu (C.T.P.); 5 Perelman School of Medicine, University of Pennsylvania, Philadelphia, PA 19104, USA; shawg@pennmedicine.upenn.edu (G.M.S.); bark@pennmedicine.upenn.edu (K.J.B.); 6 Department of Pharmacology and Molecular Sciences, Johns Hopkins University School of Medicine, Baltimore, MD 21205, USA; 7 Department of Biochemistry and Molecular Biology, Johns Hopkins University School of Medicine, Baltimore, MD 21205, USA; 8 Department of Medicine, Johns Hopkins University School of Medicine, Baltimore, MD 21287, USA; 9 Yerkes National Primate Research Center, Emory University, Atlanta, GA 30322, USA; 10 Department of Pediatrics, Weill Cornell Medicine, New York, NY 10065, USA; sallie.permar@med.cornell.edu or sap4017@med.cornell.edu (S.R.P.);; 11 Department of Biostatistics and Bioinformatics, Duke University Medical Center, Durham, NC 27710, USA; janice.mccarthy@duke.edu (J.M.M.);; 12 Department of Pediatrics, Emory University School of Medicine, Atlanta, GA 30322, USA; 13 Department of Mathematics, Pennsylvania State University, University Park, PA 16801, USA

**Keywords:** pediatric SHIV, mathematical modeling, acute SHIV infection, viral rebound, HIV cure, immune responses

## Abstract

Viral dynamics of acute HIV infection and HIV rebound following suspension of antiretroviral therapy may be qualitatively similar but must differ given, for one, the development of adaptive immune responses. Understanding the differences between acute HIV infection and viral rebound dynamics in pediatric populations may provide insights into the mechanisms of viral control with potential implications for vaccine design and the development of effective targeted therapeutics for infants and children. Mathematical models have been a crucial tool to elucidate the complex processes driving viral infections within the host. Traditionally, acute HIV infection has been modeled with the standard model of viral dynamics, initially developed to explore viral decay during treatment, while viral rebound has necessitated extensions of that standard model to incorporate explicit immune responses. Previous efforts to fit these models to viral load data have underscored differences between the two infection stages, such as increased viral clearance rate and increased death rate of infected cells during rebound. However, these findings have been predicated on viral load measurements from disparate adult individuals. In this study, we aim to bridge this gap, in infants, by comparing the dynamics of acute infection and viral rebound within the same individuals by leveraging an infant non-human primate Simian/Human Immunodeficiency Virus (SHIV) infection model. Ten infant Rhesus macaques (RMs) orally challenged with SHIV.C.CH505.375H.dCT were given ART at 8 weeks post-infection. These infants were monitored for up to 60 weeks post-infection to ensure full suppression, then underwent analytic treatment interruption (ATI), resulting in viral rebound. We use the HIV standard viral dynamics model fit to viral load measurements in a nonlinear mixed effects framework to identify primary mechanisms distinguishing acute and rebound infection dynamics. We find that the primary difference between acute infection and viral rebound is the increased death rate of infected cells during the rebound phase. These findings allow us to formulate hypotheses about the effects of adaptive immune responses. Specifically, we propose that delayed viral rebound is characterized by a stronger CD8+ T-cell response. Our analysis provides evidence to support this notion and helps elucidate the observed outcomes.

## 1. Introduction

HIV-1 remains a significant global health challenge that affected 38.4 million individuals as of 2021—including 1.7 million children—according to the World Health Organization [1,2].

The within-host viral kinetics of acute HIV infection and post-treatment interruption viral rebound in infants and children differ from each other and from patterns observed in adults. During acute infection, infants experience a rapid increase in plasma viremia, reaching levels much higher than those in adults [3,4,5]. The decline in infant plasma viremia is slow, reaching a setpoint at around 5 years of age [6,7]. Post-treatment viral rebound following analytic treatment interruption (ATI) presents a distinct profile from acute infection: while acute infection occurs in a naive immune landscape, viral rebound is dictated by the size of the established latent viral reservoir and an immune system that has been partially modulated by therapy. In this study, our objective was to determine the mechanistic differences that underlie the within-host HIV viral dynamics between acute infection and post-treatment interruption in a non-human primate model of HIV, aiming to gain insights into human pediatric HIV dynamics.

The rates of clinical progression of the disease vary between adults and children. For example, 20–30% of children undergo rapid progression to AIDS or death in the first year of life [6]. These rates of disease progression also vary depending on the age of the child [5,6]. Pediatric elite controllers are also rarer; only 0.008% of HIV-infected children are elite controllers, compared to 0.5% of adults [8,9,10]. These distinctions are mainly attributed to differences in the immunologic development of infants and the rapid expansion of CD4+ T cells associated with somatic growth [6].

Immune responses are age-dependent [6,11], establishing a highly tolerogenic and broadly anti-inflammatory environment in utero and early life [8,12] that contributes to differences in clinical outcomes between infants and adults [6]. Because of limited in utero antigen exposure, infants rely heavily on innate immunity for early protection [13,14,15,16]. While neonatal natural killer (NK) cell frequencies resemble those of adults [17,18], NK cells exhibit distinct receptor expression patterns [18,19] and reduced cytolytic activity during vertical infection [18,20,21,22,23], alongside potential memory-like adaptive traits [24,25,26,27,28,29,30,31]. Furthermore, variability in CD8+ T-cell numbers is less pronounced in infants compared to adults [32], standard neutralizing antibodies develop commonly within the first year [33], and broadly neutralizing antibodies can arise earlier, with greater breadth and potency than in adults [33,34,35,36,37,38].

Nonetheless, while infants are capable of mounting adaptive immune responses against HIV—such as CD8+ T cells detectable at birth in in utero infections [39] and polyfunctional CD8+ responses vital for pediatric elite controllers [8,10,40]—these defenses are ultimately insufficient to control viral replication [39]. Specifically, HIV-specific CD4+ T-cell responses remain infrequent and low even with early antiretroviral therapy [6,41,42], active production of HIV-specific antibody-dependent cellular cytotoxicity (ADCC) antibodies is generally delayed past 12 months of age [6,43], and non-neutralizing antibody subsets vary among non-progressors [8,44].

Mathematical modeling has been instrumental in elucidating within-host HIV dynamics, particularly in the context of viral decay following ART initiation and viral rebound after treatment interruption [45,46,47,48,49,50,51]. Various studies have used mathematical models to estimate recrudescence rates and investigate inter-individual heterogeneity in viral rebound dynamics [46,47,48]. Modeling studies in adults have examined the roles of latent reservoir size on rebound times and the reduction needed in the reservoir [45] or the strength of immune responses to achieve a cure in adult humans [52,53]. However, no prior modeling study has directly compared acute infection dynamics with post-treatment rebound dynamics within the same pediatric individuals.

To address this gap, this study aims to determine differences in the within-host HIV viral dynamics between acute infection and after treatment interruption in the same pediatric individuals. Although understanding these dynamics may be crucial to identifying viral control mechanisms and informing effective vaccine/therapeutic study design in children, performing studies in human infants is difficult. Non-human primate models of Simian Immunodeficiency Virus (SIV) and Simian/Human Immunodeficiency Virus (SHIV) show many similarities to human infections and have provided valuable insights [6,54,55,56,57]. Here, we use experimental data derived from an infant non-human primate Simian/Human Immunodeficiency Virus (SHIV) infection model that mimics breast milk HIV transmission in human infants. Infant rhesus macaques (RMs) were orally challenged with SHIV.C.CH505.375H.dCT 4 weeks after birth and ART was initiated 8 weeks after infection (wpi) and terminated a year later [58,59,60]. This model provides plasma viral load kinetics across both acute infection and post-treatment viral rebound in the same animal, and a unique opportunity to contrast dynamics.

Using a nonlinear mixed-effects (NLME) framework, we fit the standard HIV viral dynamics model [61,62], a simple and parsimonious model that accurately describes within-host dynamics [63,64,65], to viral load measurements in 10 RMs through acute infection and post-ATI phases. To capture broad kinetic shifts between acute infection and viral rebound, infection stage was treated as a covariate across all combinations of model parameters. Thus, we aim to generate hypotheses on immune mechanisms that could explain differences. Rather than selecting a single best-fit model, we analyzed an ensemble of equivalent models to identify robust shared features. Our analysis suggests that differences between acute infection and viral rebound lie in parameters that are associated with an effect of cellular immune responses, spanning both cytolytic and non-cytolytic mechanisms. Ultimately, while these findings offer translational insight into pediatric HIV, they primarily serve to generate new mechanistic hypotheses that require further experimental validation.

## 2. Methods

### 2.1. SHIV Infection Data in Infant Macaques

The data used in this study derive from a broader investigation of correlates of SHIV viral rebound in infant macaques [58,59,60]. All animals in the study were treated in accordance with Emory University and the Yerkes National Primate Research Center (YNPRC) Institutional Animal Care and Use Committee regulations (Protocol no. PROTO201700021) [58,59,60].

**Figure 1:**
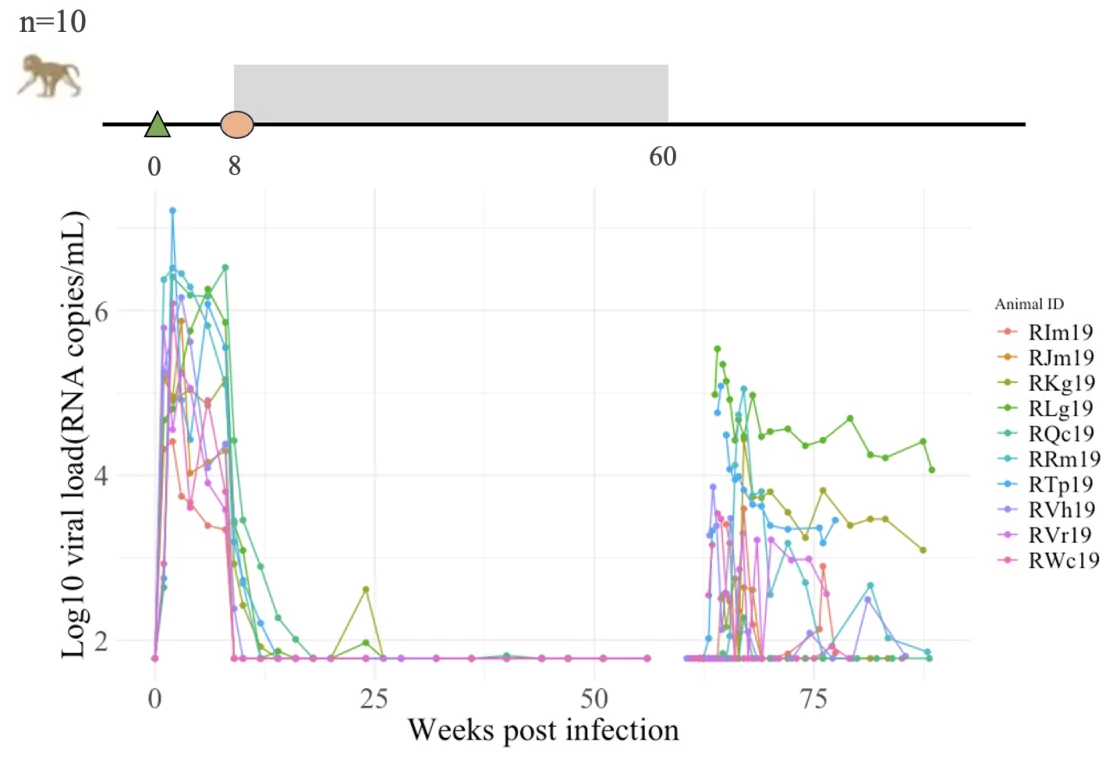
Description of the experiment. Study schematic showing ART timing (grey shaded area), as well as viral load trajectories for the 10 subjects in the experiment [60].

In brief, 30 infant Indian rhesus macaques (*Macaca mulatta*, RMs) were infected with SHIV.C.CH505.375H.dCT at 4 to 5 weeks of age via oral administration. Infant macaques were closely monitored during the pre-ART phase through weekly plasma viral load testing to capture peak viremia (Figure 1). The animals were divided into three groups of ten. Because the study objectives included assessing the impact of ART timing on viral rebound [58,59,60], the groups differed in the timing of ART initiation: 8 weeks post-infection (wpi) for “Late ART”, 2 wpi for “Intermediate ART”, and 4 or 7 days post-infection for “Early ART”.

Animals were maintained on daily antiretroviral therapy (subcutaneous dolutegravir, tenofovir, and emtricitabine) for a minimum of 52 weeks prior to interruption of treatment. Following analytic treatment interruption (ATI), animals were monitored to a common endpoint of 168 days. Longitudinal monitoring post-ATI included serial blood/tissue sampling and ImmunoPET/CT imaging to map anatomical sites of viral rebound [59]. While a limited number of gut and lymph node biopsies were collected to help identify potential sites of rebounding viremia, available tissue data were too sparse to support a mechanistic modeling analysis of viral dynamics within these specific compartments. Plasma viral loads were monitored twice weekly, approximately every 3 to 4 days, immediately following ATI to accurately capture the kinetics of viral rebound (Figure 1). The limit of detection for both pre-ART and post-ATI plasma viral assays was 60 viral copies/mL. A considerable number of viral load measurements fell below this threshold. In our analysis, these data points are treated as “censored” because they reside below the sensitivity limits of the analytical method, preventing precise quantification. In addition to viral load, the potency of antibodies in neutralizing the autologous virus envelope glycoprotein gp120 was measured via a TZM-bl assay [66] on the exact day of treatment interruption.

This study design offered us the unique opportunity to contrast acute and rebound viral kinetics in the same biological system using mathematical modeling. However, as plasma viral loads were measured weekly during the acute phase, the study collected only limited per-animal viral load data on acute infection kinetics for animals in the “Early” and “Intermediate” cohort, one and two data points per animal, respectively, which likely captured only the initial growth phase and proved insufficient for our structural model. Further, studies show that the timing of treatment initiation introduces substantial immunological differences [67,68], so pooling these cohorts risked introducing substantial and uncontrolled confounding into both our population-level estimates and individual-level variations. We therefore provide a rigorous, targeted analysis of the “Late ART” arm (10 RMs), which provides sufficient acute-phase longitudinal viral load measurements (including peak and early steady-state dynamics) for this comparison.

### 2.2. The Model

**Figure 2:**
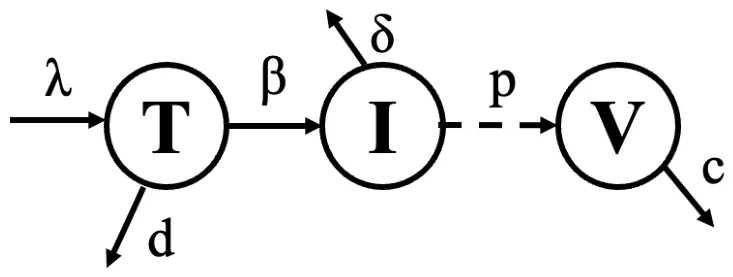
Standard viral dynamics model schematic. Schematic of the standard model. Target cells, *T* are sourced at rate λ and die at rate *d*. Virus *V* infects target cells at rate β. Infected cells, *I* die at rate δ and produce virions at rate *p*. Free virus gets cleared at rate *c*.

We employ the standard viral dynamics model, initially devised to investigate viral decay following the initiation of treatment [62]. This model, known for its ability to encapsulate within-host dynamics of HIV infection, is deliberately chosen for its simplicity and parsimony. The emphasis on a straightforward model serves as an approach to delineate the broad differences in dynamics between acute infection and viral rebound. The standard model is a target cell-limited model, i.e., HIV infection is limited by the availability of target cells, consisting of three compartments—uninfected cells, infected cells, and free virus. Target cells, *T*, are produced at rate λ, die at rate *d* and become infected at rate β. Infected cells, *I*, die at a constant rate δ and produce HIV-1 virions at rate *p*. Free virus, *V*, is cleared from the blood at rate *c* (Figure 2). This model can be expressed as the following system of differential equations:

$$\begin{array}{cc} \; & \frac{dT}{dt}=\lambda -dT-\beta TV\\ \; & \frac{dI}{dt}=\beta TV-\delta I\\ \; & \frac{dV}{dt}=pI-cV \end{array}$$

In this set of equations, *t* refers to the time (days) since treatment interruption. The description of state variables and model parameters can be found in Table 1. This target-cell limited model has been shown to capture the dynamics of early infection [63]. We focus on a simple model to characterize the general differences between the different groups. Because we are interested in broad differences between the two groups, the standard model, without incorporating explicit dynamics of adaptive immune responses, is still appropriate for capturing the dynamics of viral rebound. Adding explicit immune responses in the model would increase the number of parameters, thus adding uncertainty. The standard model exhibits both simplicity and parsimony [65].

We rescale *T* and *I* by $T_{0}$, where $T_{0}$ is the initial number of target cells per mL. This restructuring makes plain that the viral production rate *p* and $T_{0}$ are structurally unidentifiable and permits us to estimate the single composite parameter $pT_{0}$. We assume that prior to infection, target cells are at equilibrium and therefore, we set $\lambda =d\cdot T_{0}$ [63]. We also fix d=0.01 per day [63]. We estimate five parameters– mass action infectivity (β), death rate of infected cells (δ), the product of the viral production rate and initial number of target cells ($pT_{0}$), the viral clearance rate (*c*), as well as the onset of exponential viral growth ($t_{start}$), by fitting the model to plasma viral load data during acute and rebound infection.

### 2.3. Model Fitting

**Figure 3:**
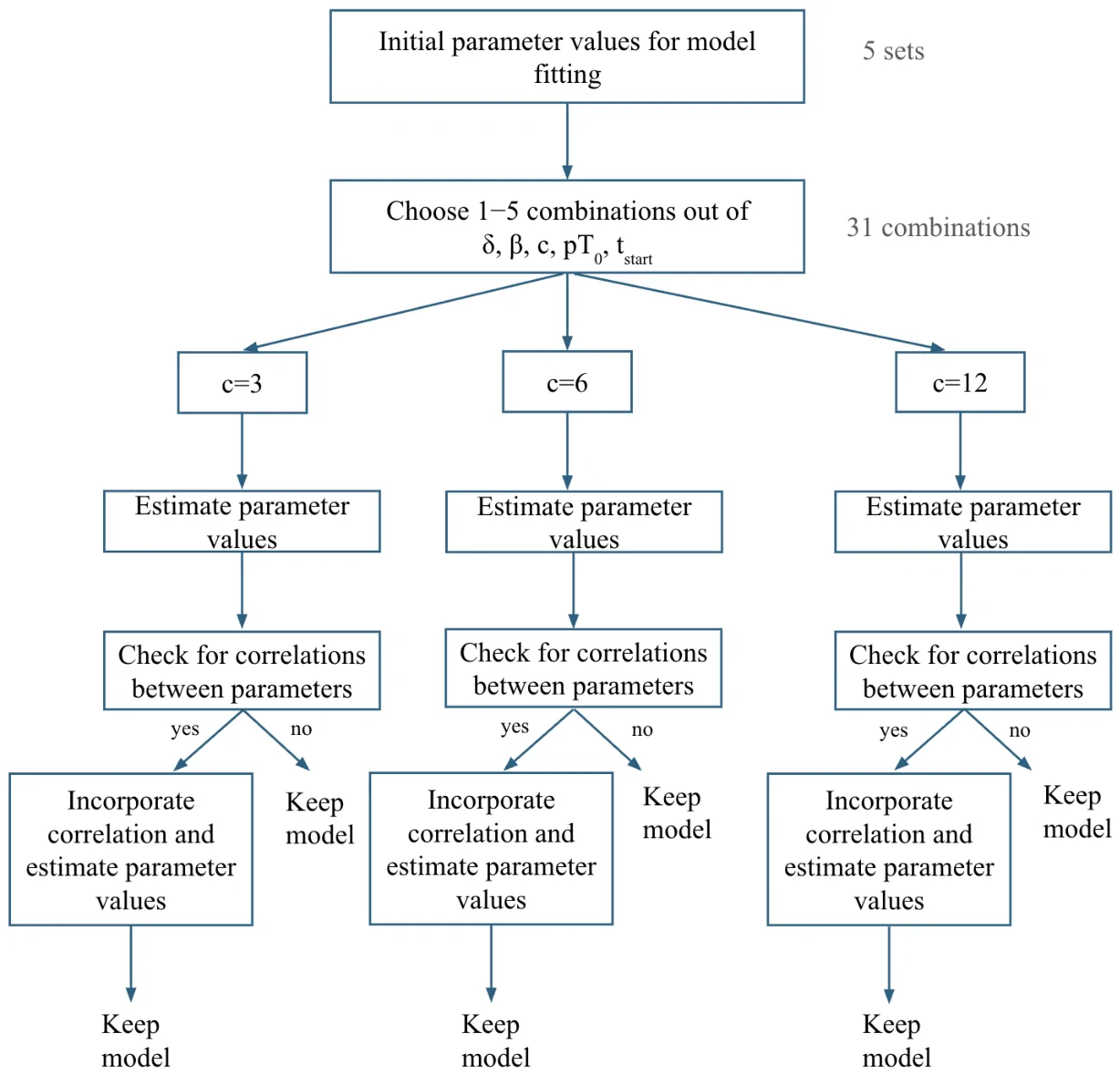
Model Fitting Schematic. Schematic of the approach we follow for model fitting and types of models tested.

We fit the standard viral dynamics to viral load measurements during acute infection before treatment initiation, and after treatment interruption. We are particularly interested in elucidating the differences between the two groups and obtain a more parsimonious model. For this reason, we use a nonlinear mixed effects (NLME) framework, as this approach allows us to capture both population-level effects, as well as individual variability. We test all models in which the stage of the infection (acute or rebound) acts as a covariate affecting all possible combinations of parameters (a total of 31 combinations). This means that for the parameter for which the stage of infection is a covariate, we assume that the values of that parameter for acute infection come from distributions with different means. We fix the population-level value of the viral clearance rate to *c* = 3, 6, and 12 ${\mathrm{day}}^{-1}$ [62,63,64], while still allowing for inter-individual variability. To better explore the parameter space, we test each model using the best initial parameter value guess along with four additional sets of initial parameter values selected randomly from an interval around the original value (Supplementary Table S1). We also add correlations between random effects in pairs of parameters when ANOVA tests indicate significance at the 0.01 significance level. Therefore, with 31 possible covariate combinations, 3 fixed values for the viral clearance rate and 5 initial sets of parameter values, we end up with 465 models to test. In addition, we test 154 models with added correlations between random effects, resulting in a total of 619 models (Figure 3). We run all models in *Monolix* through the *lixoftConnectors* package in *R*.

### 2.4. Model Selection

We do not select a single model that best fits our data. Instead, we focus on an ensemble of models that are comparably successful in explaining the observed data. Here, an ‘ensemble’ does not denote a collection of fundamentally distinct mathematical architectures. Instead, all candidate models share an identical structural backbone based on the standard differential equations of viral dynamics to minimize architectural confounding. The ensemble consists of a set of well-supported parameterizations within this single framework, where each model represents a distinct hypothesis regarding which specific parameters vary between the two infection phases. Rather than selecting a single ‘best’ model via strict information criteria, we analyzed the collective outputs of these comparably supported models to isolate biological signals that remain robust across multiple parameter combinations. Ensemble strategies are widely used in mechanistic and infectious-disease modeling, e.g., [69,70], as well as in the field of HIV immunology [71].

In addition, we do not focus on a single metric for model selection, namely the Akaike Information Criterion (AIC), which is the standard method for model selection. AIC primarily takes into account of the sampling errors in parameter estimation and can offset errors in the estimation of model parameters; the more parameters a model has, the more these methods can compensate, thus balancing model fit with model simplicity, where simplicity is defined by fewer parameters. However, when choosing a model, it is important to consider its ability to generalize predictions to different contexts. Therefore, we are interested in a more comprehensive approach, that includes the model’s predictive ability, as well as the stability of parameter estimates. We determine these models using the following criteria:

1. AIC: we consider models whose AIC is within 10 points from the lowest AIC.
2. Visual Predictive Check (VPC): The VPC is a graphical method used in statistical modeling to assess the goodness-of-fit and predictive performance of a model. It is a valuable tool for evaluating how well a model captures the observed data and how well it can predict future observations. We focus on models where the summary statistics from the observed data fall within the range of values obtained from the simulated datasets, which indicates that the model can replicate the observed data well.
3. Stability of parameter estimates: We evaluate the % rise in standard error (RSE), which is a statistical measure that quantifies the increase in the standard error of a parameter estimate when comparing two different models or conditions. The RSE is a useful metric that helps assess whether changes in the model’s complexity or data have a substantial impact on the reliability of parameter estimates. To assess the quality of a model RSE, we devise a metric titled *S* defined as the sum of RSE values above 50% divided by the number of parameters estimated.
  $$S=\frac{\sum RSE>50\%}{\mathrm{number}\mathrm{of}\mathrm{model}\mathrm{parameters}}$$
  For example, for the model estimates in Table 2, the estimated *S* value would be
  $$S=\frac{114.15+255.02+99.47}{13}=33.47$$

We first focus on models with an AIC relative to the lowest, ΔAIC<10. No additional models were excluded upon examination of the visual predictive check, as they displayed no areas of model misspecification. To narrow down our selection, we further restrict ΔAIC values, and we choose models with S<60, and ΔAIC<8 or ΔAIC<4. It should be noted that allowing for an increased *S* threshold (S<80) introduces inconsistency in the predicted association between the stage of the infection and the mean parameter values with one model predicting a decreased population value of the viral clearance rate and the onset of viral exponential growth (Supplementary Figure S1). This observation offers support for keeping *S* threshold low.

## 3. Results

**Figure 4:**
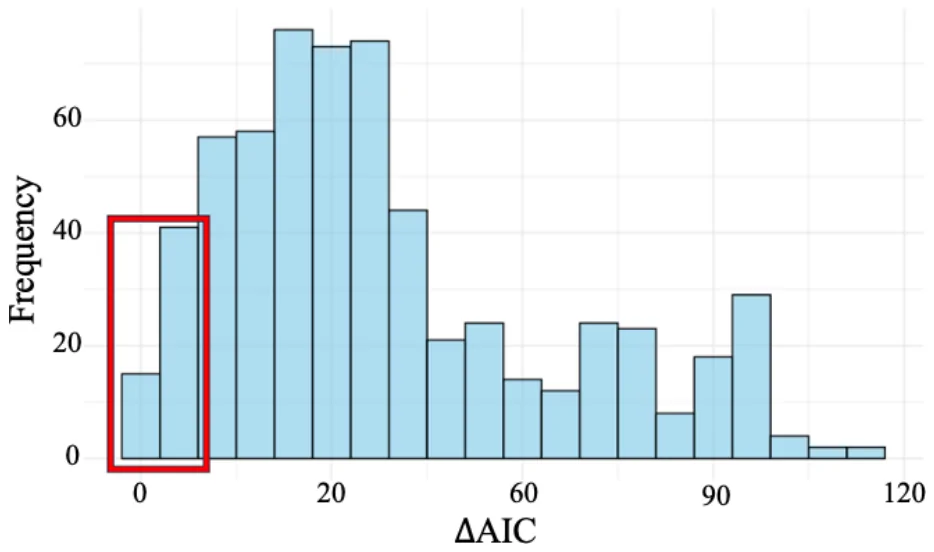
Distribution of ΔAIC. Histogram of AIC values relative to the lowest AIC of all the models tested. We reject models with ΔAIC>10. Red box indicates models with ΔAIC<10.

We fit the standard viral dynamics model to viral load measurement taken before treatment initiation (acute infection) and after treatment interruption (viral rebound) in a nonlinear mixed effects (NLME) framework. This approach results in models with varying Akaike Information Criterion (AIC) values. Initially, we focus on models with a relative AIC difference (ΔAIC) of less than 10 from the lowest AIC value, resulting in 59 models (see Figure 4). After evaluating the visual predictive check of each model, we find no signs of model misspecification, so no models are excluded at this stage. To further refine our selection, we narrow down the ΔAIC values by choosing models with an information criterion statistic S<60, and ΔAIC values of less than 8 or 4, which results in collections of 13 or 5 models, respectively. Since the results are qualitatively similar, we report the findings for the larger set of models with ΔAIC<8 below.

**Figure 5:**
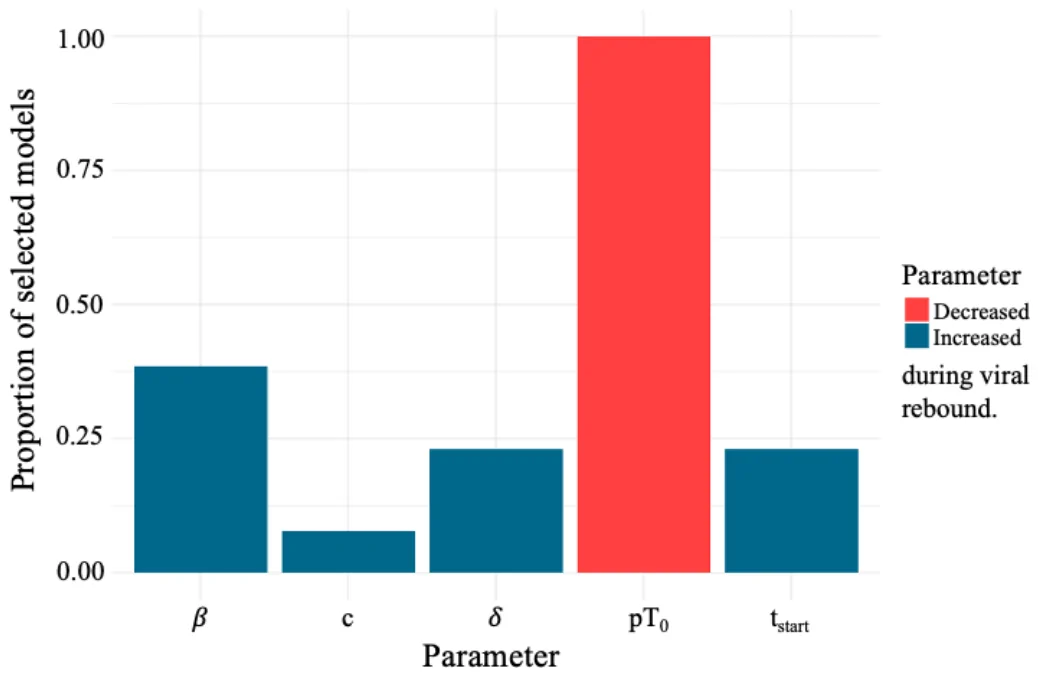
Parameters differing between acute infection and viral rebound. Bar chart depicting the proportion of times a parameter is predicted to differ between acute infection and viral rebound in all selected models. Viral clearance rate (*c*) is predicted to be different between acute infection and viral rebound in 1 out of 13 best models; death rate of infected cells (δ) and start of exponential viral growth ($t_{start}$) in 3 out 13 models; mass-action infectivity (β) in 5 out of 13 models and the product of viral production rate (*p*) and initial number of target cells ($T_{0}$) in all 13 models. Red slices signify a decrease in the value of the parameter for rebound, whereas blue-toned slices depict an increase in the rebound value of the parameter.

Table 3 provides a summary of the features of the 13 selected models. The population parameters estimated for each of the 13 models are provided in Supplementary Table S7. Below, we report the common features among selected models; for example, how many of those 13 models predict a difference in the mean value of a specific parameter between acute infection and viral rebound and whether the parameter value increases or decreases for rebound dynamics (Figure 5)? We focus on the population-level averages of our model parameters, where the two populations refer to acute infection and viral rebound. We then compare the differences in parameter values between acute infection and viral rebound and generate hypotheses for the potential mechanisms that these differences can be attributed to.

### 3.1. Exponential Viral Growth Phase Is Initiated Later in Viral Rebound

Three of the 13 selected models predict that the mean value for the onset of exponential viral growth ($t_{start}$) is slower during rebound compared to acute infection (Figure 5). This observation is expected, since the development of immune responses during rebound can reduce viremia, thus delaying rebound [72]. However, for the remaining 10 selected models, such a difference in mean values is not predicted. Instead, distributions of the onset of exponential viral growth values for acute infection and viral rebound tend to overlap (Supplementary Figure S3), pointing to the inherent heterogeneity in the stochastic phase between treatment interruption and onset of exponential viral growth.

### 3.2. Humoral Immunity Appears Underdeveloped

**Figure 6:**
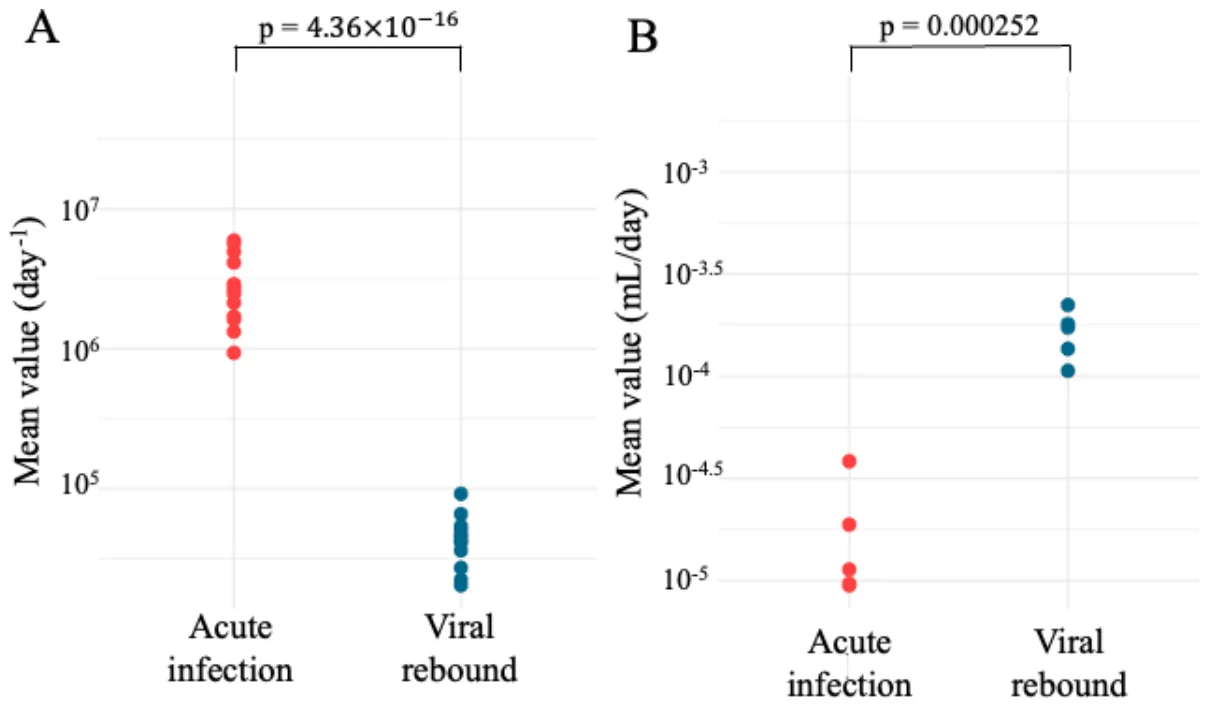
Difference in mean parameter values between viral rebound and acute infection. Mean value for the product of viral production rate and initial number of target cells, $pT_{0}$ (**A**) and mass action infectivity β (**B**), both on a $\log_{10}$ scale, for acute infection (red) and viral rebound (blue) for the 13 models selected. For both parameters, the difference in mean values is statistically significant (*t* test, *p* < 0.001).

Counter-intuitively, only 1 out of 13 models predicts a difference in mean viral clearance rate (*c*) between viral rebound and acute infection (Figure 5). Since the effects of adaptive immune responses are thought to be more pronounced during rebound dynamics, we would expect neutralizing antibodies to increase the viral clearance rate [73]. Our modeling results do not provide a strong signal for that effect. However, the distribution of viral clearance values for rebound is wider compared to acute infection and allows for values consistent with adult rebound values [52,74]. Therefore, even though the selected models predict no difference in the mean viral clearance rate, there is high heterogeneity in clearance rate rebound values (Supplementary Figure S4). The adaptive immune response mechanism that could explain increased viral clearance rate (*c*) is neutralizing antibodies (nAbs). However, we believe that this heterogeneity cannot be attributed to nAbs, since we do not observe a difference in the rebound viral clearance rate between the RMs that developed neutralizing responses and those that did not (Supplementary Figure S5). Instead, we hypothesize that the heterogeneity in the viral clearance rate may be explained by viral evolution [75]. Furthermore, 5 out of 13 models predict an increase in the viral infectivity rate by approximately one order of magnitude (Figure 6B).

### 3.3. Viral Rebound Is Characterized by a Universal Decrease in the Composite Parameter $pT_{0}$

In our ensemble of top-performing models, the most consistent parameter alteration between acute infection and viral rebound is a marked reduction in the product of the viral production rate and the initial target cell availability ($pT_{0}$). Because the viral production rate (*p*) and the initial number of target cells ($T_{0}$) are structurally unidentifiable in the standard viral dynamics model, they must be estimated as a single composite parameter. Strikingly, all 13 of the selected models predict a decrease in the population-level mean of $pT_{0}$ during the rebound phase (Supplementary Table S7; mean of $\log_{10}pT_{0}$ population estimates = 6.42 per day (sd = 0.25), decreased to 4.88 per day at rebound). This model-predicted reduction is found to be statistically significant (Figure 6A, *T*-test, p<0.001). The universal prediction of this decrease across all well-supported parameterizations suggests a robust biological difference governing the systemic capacity for viral expansion following treatment interruption, distinguishing it from the dynamics driving initial acute infection.

### 3.4. Viral Rebound Is Characterized by iNcreased Killing Rate of Infected Cells Albeit with Heterogeneity

All the selected models predict a decrease in the mean value of the product of the viral production rate and the initial number of target cells ($pT_{0}$), which are structurally unidentifiable and thus estimated as one parameter. Furthermore, for a subset of selected models (3 out of 13), the median death rate of infected cells is higher during viral rebound compared to acute infection (Figure 5). Across all 13 models, the baseline, i.e., unmodified population median death rate, is consistently $O(10^{-1})$ per day (Supplementary Table S7; mean of population medians = 0.12 per day, sd = 0.03), smaller but of the same order as those obtained in [76] for acute ${\mathrm{SHIV}}_{162P3}$. In all selected models, the distribution of the estimated death rate of infected cells is wider than for acute infection (Supplementary Figure S6), suggesting that even in these models, there are some RMs that develop these cytotoxic responses. There are multiple mechanisms that could explain an increased infected cells death rate. These include the effect of cytotoxic T lymphocytes (CTLs), NK cells, antibody-dependent cell-mediated cytotoxicity (ADCC) and antibody-dependent cell-mediated phagocytosis (ADCP). We believe that a likely explanation is CTL responses; RMs that developed neutralizing responses also developed ADCC responses, but we do not observe a difference in estimated death rate of infected cells (Supplementary Figure S7, Wilcoxon signed rank test *p*-value >0.05 for all selected models).

## 4. Discussion

In this study, we fit the standard viral dynamics model within a nonlinear mixed-effects (NLME) framework to investigate differences between acute infection and viral rebound in the same orally SHIV-infected infant RMs. Rather than focusing on the timing of viral rebound, we examine the viral dynamics after rebound. We previously investigated the role of immune responses in determining viral rebound times [72]. Here, our objective is to identify the key differences between acute infection and viral rebound as reflected by the parameters of the standard viral dynamics model and to generate hypotheses about the biological mechanisms that can underlie these differences. We use the standard viral dynamics model to provide a broad view and generate hypotheses on differences rather than focusing on specific mechanisms. We find that the main difference in the dynamics is the increase in the death rate of infected cells and the decrease in the viral production rates and the availability of target cells, pointing to the effect of cytotoxic T lymphocytes. Overall, our modeling results suggest changes in parameter values that are associated with a more pronounced cellular response compared to humoral responses.

To draw our conclusions regarding differences between acute infection and viral rebound dynamics, we adopt a novel strategy for model selection. Instead of relying solely on the Akaike Information Criterion (AIC), which is considered the standard for choosing the most parsimonious model, we opt for a diverse set of metrics and diagnostics to identify a set of optimal models. This approach allows for a more comprehensive assessment of model fits that includes model misspecification and the stability of parameter estimates. Alongside AIC, we introduce a metric called *S* to evaluate the stability of parameter estimates and utilize the Visual Predictive Check (VPC), which assesses misspecifications in structural, variability, and covariate models. Our model selection involves considering various threshold combinations for each metric (ΔAIC < 8 vs. ΔAIC < 4 and *S* < 60 vs. *S* < 80). We observe that the ΔAIC thresholds used in the analysis yield consistent effects for the change in parameter values. However, increasing the *S* threshold from 60 to 80 introduces some inconsistency: we have one selected model predicting a lower viral clearance rate for rebound and another selected model predicting a faster onset of exponential viral growth. These observations offer support for maintaining a lower threshold for our *S* metric. Thus, our model selection involves a multi-stage filtering process where candidate models are discarded if they exhibit poor parameter precision (high %RSE), lack structural stability under perturbation, or fail to adequately capture the observed data distribution during the VPC evaluation. This rigorous vetting ensures that our biological hypotheses are grounded in robust, identifiable mathematical structures rather than statistical artifacts.

We remind the reader that these findings are in a pediatric model, and infant immune systems differ from those of adults, which may shape cellular responses observed during rebound [3,4,5]. Important limitations include that data are derived from studies using a SHIV macaque model, which can lead to differences in immune responses, viral replication, and disease progression compared to HIV in humans [77], although such models also show similarities to human infections [6,54,55,56,57]. Further, animals were infected via oral inoculation. This models mother-to-child transmission through breastfeeding, and the localized mucosal immune responses may not reflect the dynamics arising from other routes of transmission such as sexual or intravenous transmission [78].

We find that even though the majority of selected models do not predict a difference in the mean viral clearance rate, the distribution for rebound is wider, allowing for values consistent with adult human rebound [52]. Furthermore, 5 out of 13 models predict an increase in the viral infectivity rate by approximately one order of magnitude (Figure 6B). This observation contrasts other experimental data [79] and mathematical modeling results [80]. We speculate that the increased viral infectivity rate during rebound could be attributed to the increased proportion of target cells that can be easily infected. HIV-specific activated CD4+ T cells are the preferential target of infection, and undergo a clonal expansion due to stimulation by the virus [81,82,83]. This means that at later stages, there is a larger proportion of target cells that can be easily infected, thus increasing the viral infectivity rate. In addition, all of the selected models predict a decrease in the mean value of the product of the viral production rate and the initial number of target cells. Since the viral production rate and the initial number of target cells are not identifiable, we cannot determine which parameter accounts for this decrease. We hypothesize that either parameter decreases. Regarding the initial number of target cells, in pediatric HIV, CD4+ T cells do not recover to pre-infection levels under ART [8,84,85], which could lead to a decreased rebound value. Viral production rate could also be lower at later stages of the infection due to the non-cytolytic effects of CD8+ T cells.

Our results point to the importance of CD8+ T-cell responses [39,86] and indicate the development of both cytolytic and non-cytolytic effects during rebound. We observe an increase in the killing rate of infected cells, although the estimated death rate is generally lower compared to the adult rebound values [52,87] and the variability among rebounding RMs, some of whom display values consistent with acute infection (Supplementary Figure S5). In addition, we find a decrease in the product of scaled viral production rate, which can be attributed to a reduced viral production due to non-cytolytic effects of CD8+ T cells [71]. Recent modeling works also provide support for a dual function of CD8+ T cells in HIV-1 infection [71]. Indeed, CD8+ T-cell responses are a hallmark of control. For instance, in pediatric, as well as adult, cases, elite controllers display a higher frequency of HIV Gag-specifc, polyfunctional CD8+ T-cell responses capable of cytokine release compared to progressors [8,88,89]. Experimental findings also support this observation: CD8+ T-cell depletion in non-human primate models of HIV/SIV/SHIV result in a more profound increase in viral replication in elite controllers [90], followed by a recovery of viral control once CD8+ T-cell population is restored [8,91]. CD8+ T-cell responses can also differentiate between rebounders and controllers in SIV-infected macaques, with individuals able to control the infection displaying a long-term memory CD8+ T-cell response with enhanced suppressive activity [92].

However, despite the importance of CD8+ T cells in HIV control, their mechanism of action remains poorly understood [93]. Evidence points to a non-cytolytic effect of CD8+ T cells: two groups examined the duration of SIV-infected cell lifespan following treatment with nucleotide reverse transcriptase inhibitors (NRTIs), with or without the presence of CD8+ cells, finding no difference in average cell lifespan [94,95]. Subsequent studies have both supported [96,97,98,99] and challenged this effect pointing to cytotoxic activity [100,101]. More recently, mathematical modeling of data derived from SIV-infected macaques treated with the integrase inhibitor ralegravir, in the presence or absence of CD8+ cells revealed a dual effect: before viral integration, the half-life on infected cells was shorter for the group with CD8+ cells compared to the group whose CD8+ cells were depleted. Furthermore, viral production rates were higher in the absence of CD8+ cells. These results suggest that CD8+ cells have both a non-cytolytic effect in suppressing viral production, as well as a cytolytic effect before viral integration [71]. Recent studies in vitro have also pointed to a non-cytolytic effect of CD8+ T cells by preventing viral production rather than viral infectivity, which is consistent with Policicchio et al. [71], as well as with our observation for a tendency toward increased infectivity during rebound [102].

An alternative hypothesis that could explain the increased killing rate of infected cells is natural killer (NK) cell activity. NK cells have been shown to have an antiviral effect in HIV infection [103] and may play an important role in the outcome of the infection [104,105,106]. Although considered as nonspecific, innate effector cells, there is growing evidence that supports adaptive, memory-like features of NK cells [24]. In humans, this subset of long-lived NK-cells with memory-like features has been shown to exhibit increased effector functions upon re-encountering viral antigens or proper activation with pro-inflammatory cytokines [25,26,27,28,29,30]. The presence of antigen-specific NK cells has also been reported in rhesus macaques infected with SIV/SHIV. These cells exhibit specificity towards gag- and env-viral proteins loaded on autologous DCs [107]. Notably, this response appears to be long-lasting, since animals vaccinated with an adenovirus 26-vectored SIV vaccine 5 years prior still showed a recall of these adaptive traits in NK cells, associated with a high degree of cytotoxicity. Nevertheless, this observation lacks direct evidence of NK cell memory responses in humans [108]. In humans, the temporal efficacy of NK cell activity during HIV infection remains not well understood. Some studies have shown increased cytolytic activity of NK cells during HIV infection [109]. Even though HIV-1 infection initially causes a substantial decrease in the NK cell population, the function of those NK cells is enhanced during viremic infection, compared to treated and suppressed individuals or negative controls [110]. However, it has also been shown that NK cells upregulate inhibitory receptors during viremic HIV infection, affecting the killing ability of NK cells [111,112], as well as causing sustained impairment [113]. Considering the importance of innate responses in infant immunity [13,16,31], as well as a bias of Th2 responses in infants [114], the ability of NK cells to contribute to cytolytic effects during pediatric HIV infection is a valid hypothesis that should be further investigated.

It should be noted that the differences we find between acute infection and viral rebound are associations with parameter values. Even though this provides a simple framework to explore patterns in the relationship between the stage of the infection and key viral kinetics parameters, the mechanisms that account for those relationships may sometimes be difficult to disentangle. For example, we find increased death rate of infected cells, which we attribute to CD8+ T-cell responses. However, NK cells also have cytotoxic effects and provide an alternative explanation. Further, there is a growing body of evidence that suggests that CD4+ T cells also have a direct killing effect [115,116]. An important future direction would include developing models that incorporate more explicit mechanisms, along with longitudinal measurements of the compartments of interest to further elucidate these dynamics.

Our ability to confirm mechanisms underlying these parameter shifts is also constrained by data availability. Our dataset lacked longitudinal CD4+ and CD8+ T-cell counts or functional kinetics that may have helped us, for example, decouple inferred changes in infected cell death rates from general target cell depletion or early neutralizing antibody effects. Future studies incorporating concurrent, high-resolution cellular immunology metrics would be highly beneficial to explore the hypotheses generated by our mathematical framework.

Although we find that only one of the selected models predicts a difference in mean viral clearance, the distribution of individual values for rebound is wider and includes values consistent with the rebound of adult HIV infections [52,74]. Counterintuitively, this difference is not attributed to neutralizing antibody responses but to innate responses, since the distribution of parameter values does not differ based on whether the RMs had developed neutralizing antibodies (Supplementary Figures S3 and S4). The role of humoral responses in viral control in infants is poorly examined [8]. In adults, the frequency of HIV-specific memory B cells in elite controllers is higher compared to individuals on ART [8,117]. Although this has not been examined in pediatric ECs, B cell dysfunction appears to be a distinctive feature of HIV disease advancement in children [8,118]. In addition to neutralization, antibodies capable of antibody-dependent cell-mediated cytotoxicity (ADCC) have been identified in pediatric non-progressors [8,119], as well as adult ECs. However, we do not observe differences in viral clearance rate among RMs based on whether they had developed antibodies capable of ADCC activity.

In addition to identifying key differences between acute infection and viral rebound, our modeling framework provides a quantitative roadmap for future experimental designs by identifying the key kinetic parameters—specifically the variations in target cell availability, viral production, and infected cell clearance rates—that govern viral rebound. Although direct measurements of cellular immune components or cytokine profiles were not available in this prospective cohort, these modeling insights highlight that longitudinal tracking of cytotoxic T lymphocyte (CTL) activation and tissue-specific target cell dynamics should be prioritized in future studies to validate these predicted mechanisms.

## Figures and Tables

**Table 1:** Baseline model state variables and parameters description.

Parameter	Meaning	Unit
*T*	Target cells	cells/mL
*I*	Infected cells	cells/mL
*V*	Plasma free virus	copies/mL
$T_{0}$	Initial number of target cells	cells/mL
λ	Target cell production	cells/mL/day
*d*	Target cell death rate	${\mathrm{day}}^{-1}$
β	Mass-action infectivity	mL/day
δ	Infected cell death rate	${\mathrm{day}}^{-1}$
*p*	Viral production rate	${\mathrm{day}}^{-1}$
*c*	Virion clearance rate	${\mathrm{day}}^{-1}$
$t_{\mathrm{start}}$	Start of exponential viral growth	days

**Table 2:** Parameter values: estimates and rise in standard error (RSE) for one of the models selected (also Supplementary Figure S2). In this model we fix the mean viral clearance rate to *c* = 3 ${\mathrm{day}}^{-1}$ for acute infection, and we assume that the product of viral production rate and initial number of target cells ($pT_{0}$) as well as the mass action infectivity (β) are different between acute and rebound and add a correlation between *c* and β. RSE values between 50 and 100% are shown in blue, values between 100 and 200% are shown in orange, and values above 200% are shown in red.

	Value	RSE%
Fixed effects		
δ	0.17	32.78
$\log_{10}\left(pT_{0}\right)$	6.41	3.57
Rand. eff. of rebound covariate on $pT_{0}$	−2.1	22.77
*c*	3	
$\log_{10}\left(\beta \right)$	−4.94	11.17
Rand. eff. of rebound covariate on $\log_{10}\left(\beta \right)$	1.18	−16.92
$t_{\mathrm{start}}$	3.11	114.15
Standard deviation of the random effects		
δ	0.86	12.07
$\log_{10}\left(pT_{0}\right)$	0.21	14.11
*c*	1.9	255.02
$\log_{10}\left(\beta \right)$	0.47	27.77
$t_{\mathrm{start}}$	0.8	99.47
Correlations		
$c-\log_{10}\left(\beta \right)$	0.92	11.27
Error model parameters		
*a*	0.58	4.57

**Table 3:** Summary of selected models. For each of the selected models, the light purple-colored cells signify the parameters for which we assume a difference in the mean value between acute infection and viral rebound. We also report the mean viral clearance rate *c*, as well as the parameters for which we incorporate correlations between random effects.

Model	Mass Action	Viral Clearance	Death Rate of	Viral Prod. Rate	Onset of Exp.	Correlations
Code	Infectivity	Rate (${\mathrm{Day}}^{-1}$)	Infected Cells	× **Initial Target**	Viral Growth	Between
	(mL/Day), β	c	(${\mathrm{Day}}^{-1}$), δ	Cell Number, ${\mathrm{pT}}_{0}$	(Days), $t_{\mathrm{Start}}$	Random Effects
12E		3				
36DC1		12				c−β
32EC1		12				c−β
32A		12				
112BC1		3				c−β
111AC1		3				c−β
22EC1		6				c−β
16AC1		3				c−β
211DC1		6				c−β
324AC1		12				c−β
26D		6				
324DC1		12				c−β
122CC1		3				c−β

## Data Availability

The data utilized in this study originates from experimental research. For access to these data, please contact the Principal Investigator of the study, Dr. Ann Chahroudi (ann.m.chahroudi@emory.edu), directly. All analysis code, along with a simulated test dataset are available at https://github.com/elliemainou/reboundP01-AcRe, accessed on 8 August 2026.

## Supplementary Materials

The following supporting information can be downloaded at https://www.mdpi.com/article/10.3390/v18101076/s1, Figure S1: Bar chart depicting the proportion times of a parameter is predicted to differ between acute infection and viral rebound in all selected models with ΔAIC<8 and S<80; Figure S2: Example of one of the selected models selected; Figure S3: Distribution of the onset of exponential viral growth ($t_{start}$) for acute infection (red) and viral rebound (blue) for all selected models; Figure S4: Distribution of the estimated viral clearance rate (c) for acute infection (red) and viral rebound (blue) for all selected models; Figure S5: Scatterplot of the estimated viral clearance rate per day (c) for acute infection (red) and viral rebound (blue) for all selected models based on whether the RM developed antibody neutralizing responses; Figure S6: Distribution of the estimated infected cell death rate (δ) for acute infection (red) and viral rebound (blue) for all selected models; Figure S7: Scatterplot of the estimated death rate of infected cells per day (δ) for acute infection (red) and viral rebound (blue) for all selected models based on whether the RM developed antibody neutralizing responses; Table S1: Initial parameter values used for model fitting; Table S2: Summary of model fits; Table S3: Estimated parameter values for each RM; Table S4: List of initially selected models; Table S5: Listed of selected models for each type of model selection; Table S6: Estimated parameter values and relative standard error for one of the selected models; Table S7: Summary of the 13 best models.

## References

[1] WHO HIV. World Health Organization 2022. http://www.who.int/data/gho/data/themes/HIV-AIDS.

[2] UNAIDS (2021). Global HIV & AIDS Statistics—Fact Sheet. http://www.unaids.org/en/resources/fact-sheet.

[3] European Collaborative Study (2002). Level and pattern of HIV-1-RNA viral load over age: Differences between girls and boys?. AIDS.

[4] Martinez D.R., Permar S.R., Fouda G.G. (2016). Contrasting adult and infant immune responses to HIV infection and vaccination. Clin. Vaccine Immunol..

[5] Dunn D. (2003). HIV Paediatric Prognostic Markers Collaborative Study Group: Short-term risk of disease progression in HIV-1-infected children receiving no antiretroviral therapy or zidovudine monotherapy: A meta-analysis. Lancet.

[6] Tobin N.H., Aldrovandi G.M. (2013). Immunology of pediatric HIV infection. Immunol. Rev..

[7] McIntosh K., Shevitz A., Zaknun D., Kornegay J., Chatis P., Karthas N., Burchett S.K. (1996). Age-and time-related changes in extracellular viral load in children vertically infected by Human Immunodeficiency Virus. Pediatr. Infect. Dis. J..

[8] Berendam S.J., Nelson A.N., Yagnik B., Goswami R., Styles T.M., Neja M.A., Phan C.T., Dankwa S., Byrd A.Y., Garrido C. (2022). Challenges and Opportunities of Therapies Targeting Early Life Immunity for Pediatric HIV Cure. Front. Immunol..

[9] Vieira V.A., Zuidewind P., Muenchhoff M., Roider J., Millar J., Clapson M., Van Zyl A., Shingadia D., Adland E., Athavale R. (2019). Strong sex bias in elite control of paediatric HIV infection. AIDS.

[10] Vieira V.A., Millar J., Adland E., Muenchhoff M., Roider J., Guash C.F., Peluso D., Thomé B., Garcia-Guerrero M.C., Puertas M.C. (2022). Robust HIV-specific CD4+ and CD8+ T-cell responses distinguish elite control in adolescents living with HIV from viremic nonprogressors. AIDS.

[11] Adkins B., Leclerc C., Marshall-Clarke S. (2004). Neonatal adaptive immunity comes of age. Nat. Rev. Immunol..

[12] Berendam S.J., Morgan-Asiedu P.K., Mangan R.J., Li S.H., Heimsath H., Luo K., Curtis A.D., Eudailey J.A., Fox C.B., Tomai M.A. (2021). Different adjuvanted pediatric HIV envelope vaccines induced distinct plasma antibody responses despite similar B cell receptor repertoires in infant Rhesus macaques. PLoS ONE.

[13] Levy O. (2007). Innate immunity of the newborn: Basic mechanisms and clinical correlates. Nat. Rev. Immunol..

[14] Firth M.A., Shewen P.E., Hodgins D.C. (2005). Passive and active components of neonatal innate immune defenses. Anim. Health Res. Rev..

[15] Krishnan S., Craven M., Welliver R.C., Ahmad N., Halonen M. (2003). Differences in participation of innate and adaptive immunity to respiratory syncytial virus in adults and neonates. J. Infect. Dis..

[16] Ku C.L., Von Bernuth H., Picard C., Zhang S.Y., Chang H.H., Yang K., Chrabieh M., Issekutz A.C., Cunningham C.K., Gallin J. (2007). Selective predisposition to bacterial infections in IRAK-4–deficient children: IRAK-4–dependent TLRs are otherwise redundant in protective immunity. J. Exp. Med..

[17] Nakazawa T., Agematsu K., Yabuhara A. (1997). Later development of Fas ligand-mediated cytotoxicity as compared with granule-mediated cytotoxicity during the maturation of natural killer cells. Immunology.

[18] Muenchhoff M., Prendergast A.J., Goulder P.J.R. (2014). Immunity to HIV in early life. Front. Immunol..

[19] Sundström Y., Nilsson C., Lilja G., Kärre K., Troye-Blomberg M., Berg L. (2007). The expression of human natural killer cell receptors in early life. Scand. J. Immunol..

[20] Ballan W.M., Vu B.A.N., Long B.R., Loo C.P., Michaëlsson J., Barbour J.D., Lanier L.L., Wiznia A.A., Abadi J., Fennelly G.J. (2007). Natural killer cells in perinatally HIV-1-infected children exhibit less degranulation compared to HIV-1-exposed uninfected children and their expression of KIR2DL3, NKG2C, and NKp46 correlates with disease severity. J. Immunol..

[21] Ziegner U., Campbell D., Weinhold K., Frank I., Rutstein R., Starr S.E. (1999). Deficient antibody-dependent cellular cytotoxicity against Human Immunodeficiency Virus (HIV)-expressing target cells in perinatal HIV infection. Clin. Diagn. Lab. Immunol..

[22] Yabuhara A., Kawal H., Komiyama A. (1990). Development of natural killer cytotoxicity during childhood: Marked increases in number of natural killer cells with adequate cytotoxic abilities during infancy to early childhood. Pediatr. Res..

[23] Dalle J.H., Menezes J., Wagner É., Blagdon M., Champagne J., Champagne M.A., Duval M. (2005). Characterization of cord blood natural killer cells: Implications for transplantation and neonatal infections. Pediatr. Res..

[24] Paust S., Von Andrian U.H. (2011). Natural killer cell memory. Nat. Immunol..

[25] Brunetta E., Hudspeth K.L., Mavilio D. (2010). Pathologic natural killer cell subset redistribution in HIV-1 infection: New insights in pathophysiology and clinical outcomes. J. Leukoc. Biol..

[26] Lopez-Vergès S., Milush J.M., Schwartz B.S., Pando M.J., Jarjoura J., York V.A., Houchins J.P., Miller S., Kang S.M., Norris P.J. (2011). Expansion of a unique CD57+ NKG2Chi natural killer cell subset during acute human cytomegalovirus infection. Proc. Natl. Acad. Sci. USA.

[27] Romee R., Schneider S.E., Leong J.W., Chase J.M., Keppel C.R., Sullivan R.P., Cooper M.A., Fehniger T.A. (2012). Cytokine activation induces human memory-like NK cells. Blood.

[28] Della Chiesa M., Sivori S., Carlomagno S., Moretta L., Moretta A. (2015). Activating KIRs and NKG2C in viral infections: Toward NK cell memory?. Front. Immunol..

[29] Wu Z., Sinzger C., Frascaroli G., Reichel J., Bayer C., Wang L., Schirmbeck R., Mertens T. (2013). Human cytomegalovirus-induced NKG2Chi CD57hi natural killer cells are effectors dependent on humoral antiviral immunity. J. Virol..

[30] Djaoud Z., David G., Bressollette C., Willem C., Rettman P., Gagne K., Legrand N., Mehlal S., Cesbron A., Imbert-Marcille B.M. (2013). Amplified NKG2C+ NK cells in cytomegalovirus (CMV) infection preferentially express killer cell Ig-like receptor 2DL: Functional impact in controlling CMV-infected dendritic cells. J. Immunol..

[31] Dowling D.J., Levy O. (2014). Ontogeny of early life immunity. Trends Immunol..

[32] Krogstad P., Uittenbogaart C.H., Dickover R., Bryson Y.J., Plaeger S., Garfinkel A. (1999). Primary HIV infection of infants: The effects of somatic growth on lymphocyte and virus dynamics. Clin. Immunol..

[33] Goo L., Chohan V., Nduati R., Overbaugh J. (2014). Early development of broadly neutralizing antibodies in HIV-1-infected infants. Nat. Med..

[34] Mishra N., Sharma S., Dobhal A., Kumar S., Chawla H., Singh R., Makhdoomi M.A., Das B.K., Lodha R., Kabra S.K. (2020). Broadly neutralizing plasma antibodies effective against autologous circulating viruses in infants with multivariant HIV-1 infection. Nat. Commun..

[35] Muenchhoff M., Adland E., Karimanzira O., Crowther C., Pace M., Csala A., Leitman E., Moonsamy A., McGregor C., Hurst J. (2016). Nonprogressing HIV-infected children share fundamental immunological features of nonpathogenic SIV infection. Sci. Transl. Med..

[36] Ditse Z., Muenchhoff M., Adland E., Jooste P., Goulder P., Moore P.L., Morris L. (2018). HIV-1 subtype C-infected children with exceptional neutralization breadth exhibit polyclonal responses targeting known epitopes. J. Virol..

[37] Ghulam-Smith M., Olson A., White L.F., Chasela C.S., Ellington S.R., Kourtis A.P., Jamieson D.J., Tegha G., Van Der Horst C.M., Sagar M. (2017). Maternal but not infant anti-HIV-1 neutralizing antibody response associates with enhanced transmission and infant morbidity. mBio.

[38] Makhdoomi M.A., Khan L., Kumar S., Aggarwal H., Singh R., Lodha R., Singla M., Das B.K., Kabra S.K., Luthra K. (2017). Evolution of cross-neutralizing antibodies and mapping epitope specificity in plasma of chronic HIV-1-infected antiretroviral therapy-naïve children from India. J. Gen. Virol..

[39] Thobakgale C.F., Ramduth D., Reddy S., Mkhwanazi N., de Pierres C., Moodley E., Mphatswe W., Blanckenberg N., Cengimbo A., Prendergast A. (2007). Human Immunodeficiency Virus-specific CD8+ T-cell activity is detectable from birth in the majority of in utero-infected infants. J. Virol..

[40] Yan J., Sabbaj S., Bansal A., Amatya N., Shacka J.J., Goepfert P.A., Heath S.L. (2013). HIV-specific CD8+ T cells from elite controllers are primed for survival. J. Virol..

[41] Ramduth D., Thobakgale C.F., Mkhwanazi N.P., De Pierres C., Reddy S., Van Der Stok M., Mncube Z., Mphatswe W., Blanckenberg N., Cengimbo A. (2008). Detection of HIV Type 1 gag-specific CD4+ T cell responses in acutely infected infants. AIDS Res. Hum. Retroviruses.

[42] Luzuriaga K., McManus M., Catalina M., Mayack S., Sharkey M., Stevenson M., Sullivan J.L. (2000). Early therapy of vertical Human Immunodeficiency Virus Type 1 (HIV-1) infection: Control of viral replication and absence of persistent HIV-1-specific immune responses. J. Virol..

[43] Pugatch D., Sullivan J.L., Pikora C.A., Luzuriaga K., Group W.S. (1997). Delayed generation of antibodies mediating Human Immunodeficiency Virus Type 1-specific antibody-dependent cellular cytotoxicity in vertically infected infants. J. Infect. Dis..

[44] Muenchhoff M., Chung A., Roider J., Dugast A.S., Richardson S., Kløverpris H., Leslie A., Ndung’u T., Moore P., Alter G. (2020). Distinct Immunoglobulin Fc Glycosylation Patterns Are Associated with Disease Nonprogression and Broadly Neutralizing Antibody Responses in Children with HIV Infection. mSphere.

[45] Hill A.L., Rosenbloom D.I., Fu F., Nowak M.A., Siliciano R.F. (2014). Predicting the outcomes of treatment to eradicate the latent reservoir for HIV-1. Proc. Natl. Acad. Sci. USA.

[46] Hill A.L., Rosenbloom D.I., Goldstein E., Hanhauser E., Kuritzkes D.R., Siliciano R.F., Henrich T.J. (2016). Real-time predictions of reservoir size and rebound time during antiretroviral therapy interruption trials for HIV. PLoS Pathog..

[47] Pinkevych M., Cromer D., Tolstrup M., Grimm A.J., Cooper D.A., Lewin S.R., Søgaard O.S., Rasmussen T.A., Kent S.J., Kelleher A.D. (2015). HIV reactivation from latency after treatment interruption occurs on average every 5–8 days—Implications for HIV remission. PLoS Pathog..

[48] Mykola P., J K.S., Martin T., R L.S., A C.D., S S.O., A R.T., D K.A., Deborah C., P D.M. (2016). Modeling of experimental data supports HIV reactivation from latency after treatment interruption on average once every 5–8 days. PLoS Pathog..

[49] Fennessey C.M., Pinkevych M., Immonen T.T., Reynaldi A., Venturi V., Nadella P., Reid C., Newman L., Lipkey L., Oswald K. (2017). Genetically-barcoded SIV facilitates enumeration of rebound variants and estimation of reactivation rates in nonhuman primates following interruption of suppressive antiretroviral therapy. PLoS Pathog..

[50] Conway J.M., Perelson A.S., Li J.Z. (2019). Predictions of time to HIV viral rebound following ART suspension that incorporate personal biomarkers. PLoS Comput. Biol..

[51] Conway J.M., Meily P., Li J.Z., Perelson A.S. (2021). Unified model of short- and long-term HIV viral rebound for clinical trial planning. J. R. Soc. Interface.

[52] Conway J.M., Perelson A.S. (2015). Post-treatment control of HIV infection. Proc. Natl. Acad. Sci. USA.

[53] Desikan R., Raja R., Dixit N.M. (2020). Early exposure to broadly neutralizing antibodies may trigger a dynamical switch from progressive disease to lasting control of SHIV infection. PLoS Comput. Biol..

[54] Abel K. (2009). The Rhesus macaque pediatric SIV infection model-a valuable tool in understanding infant HIV-1 pathogenesis and for designing pediatric HIV-1 prevention strategies. Curr. HIV Res..

[55] Jayaraman P., Mohan D., Polacino P., Kuller L., Sheikh N., Bielefeldt-Ohmann H., Richardson B., Anderson D., Hu S.L., Haigwood N.L. (2004). Perinatal transmission of SHIV-SF162P3 in Macaca nemestrina. J. Med. Primatol..

[56] McClure H., Anderson D., Ansari A., Klumpp S. (1992). The Simian Immunodeficiency Virus infected macaque: A model for pediatric AIDS. Pathol. Biol..

[57] Jayaraman P., Zhu T., Misher L., Mohan D., Kuller L., Polacino P., Richardson B.A., Bielefeldt-Ohmann H., Anderson D., Hu S.L. (2007). Evidence for persistent, occult infection in neonatal macaques following perinatal transmission of simian-Human Immunodeficiency Virus SF162P3. J. Virol..

[58] Obregon-Perko V., Bricker K.M., Mensah G., Uddin F., Kumar M.R., Fray E.J., Siliciano R.F., Schoof N., Horner A., Mavigner M. (2020). Simian-Human Immunodeficiency Virus SHIV.C.CH505 Persistence in ART-suppressed Infant Macaques Is Characterized by Elevated SHIV RNA in the Gut and a High Abundance of Intact SHIV DNA in Naive CD4+ T Cells. J. Virol..

[59] Obregon-Perko V., Bricker K.M., Mensah G., Uddin F., Rotolo L., Vanover D., Desai Y., Santangelo P.J., Jean S., Wood J. (2021). Dynamics and origin of rebound viremia in SHIV-infected infant macaques following interruption of long-term ART. JCI Insight.

[60] Obregon-Perko V., Awasthi A., Barfield R., Berendam S.J., Yagnik B., Styles T., Tharp G., Gupta P.M., Mensah G., Neja M. (2025). Identifying correlates of viral rebound timing and viral control in SHIV-infected infant macaques after ART interruption. Sci. Transl. Med..

[61] Perelson A.S., Neumann A.U., Markowitz M., Leonard J.M., Ho D.D. (1996). HIV-1 dynamics in vivo: Virion clearance rate, infected cell life-span and viral generation time. Science.

[62] Perelson A.S., Essunger P., Cao Y., Vesanen M., Hurley A., Saksela K., Markowitz M., Ho D.D. (1997). Decay characteristics of HIV-1-infected compartments during combination therapy. Nature.

[63] Stafford M.A., Corey L., Cao Y., Daar E.S., Ho D.D., Perelson A.S. (2000). Modeling plasma virus concentration during primary HIV infection. J. Theor. Biol..

[64] Reeves D., Rolland M., Dearlove B., Li Y., Robb M., Schiffer J., Gilbert P., Cardozo-Ojeda E., Mayer B. (2021). Timing HIV infection with a simple and accurate population viral dynamics model. J. R. Soc. Interface.

[65] Mainou E., Ribeiro R.M., Conway J.M. (2024). Modeling dynamics of acute HIV infection incorporating density-dependent cell death and multiplicity of infection. PLoS Comput. Biol..

[66] Sarzotti-Kelsoe M., Bailer R., Turk E., Lin C., Bilska M., Greene K., Gao H., Todd C., Ozaki D., Seaman M. (2014). Optimization and validation of the TZM-bl assay for standardized assessments of neutralizing antibodies against HIV-1. J. Immunol. Methods.

[67] Li J.Z., Melberg M., Kittilson A., Abdel-Mohsen M., Li Y., Aga E., Bosch R.J., Wonderlich E.R., Kinslow J., Sharaf L.B. (2024). Predictors of HIV rebound differ by timing of antiretroviral therapy initiation. JCI Insight.

[68] Gunst J.D., Gohil J., Li J.Z., Bosch R.J., White A., Seamon C., Chun T.W., Mothe B., Gittens K., Praiss L. (2025). Time to HIV viral rebound and frequency of post-treatment control after analytical interruption of antiretroviral therapy: An individual data-based meta-analysis of 24 prospective studies. Nat. Commun..

[69] Shea K., Borchering R.K., Probert W.J.M., Howerton E., Bogich T.L., Li S.L., van Panhuis W.G., Viboud C., Aguás R., Belov A.A. (2023). Multiple models for outbreak decision support in the face of uncertainty. Proc. Natl. Acad. Sci. USA.

[70] Howerton E., Contamin L., Mullany L.C., Qin M., Reich N.G., Bents S., Borchering R.K., mok Jung S., Loo S.L., Smith C.P. (2023). Evaluation of the US COVID-19 Scenario Modeling Hub for informing pandemic response under uncertainty. Nat. Commun..

[71] Policicchio B.B., Cardozo-Ojeda E.F., Xu C., Ma D., He T., Raehtz K.D., Sivanandham R., Kleinman A.J., Perelson A.S., Apetrei C. (2023). CD8+ T cells control SIV infection using both cytolytic effects and non-cytolytic suppression of virus production. Nat. Commun..

[72] Mainou E., Berendam S., Obregon-Perko V., Uffman E., Phan C., GM S., Bar K., Kumar M., Fray E., Siliciano J. (2024). Assessing the impact of autologous neutralizing antibodies on viral rebound in postnatally SHIV-infected ART-treated infant Rhesus macaques. Epidemics.

[73] Igarashi T., Brown C., Azadegan A., Haigwood N., Dimitrov D., Martin M.A., Shibata R. (1999). Human Immunodeficiency Virus Type 1 neutralizing antibodies accelerate clearance of cell–free virions from blood plasma. Nat. Med..

[74] Ramratnam B., Bonhoeffer S., Binley J., Hurley A., Zhang L., Mittler J.E., Markowitz M., Moore J.P., Perelson A.S., Ho D.D. (1999). Rapid production and clearance of HIV-1 and hepatitis C virus assessed by large volume plasma apheresis. Lancet.

[75] Boutwell C.L., Rolland M.M., Herbeck J.T., Mullins J.I., Allen T.M. (2010). Viral evolution and escape during acute HIV-1 infection. J. Infect. Dis..

[76] Ruian K., Mian-er C., David L., Gerardo G.L.J., S P.A. (2017). On the Death Rate of Abortively Infected Cells: Estimation from Simian-Human Immunodeficiency Virus Infection. J. Virol..

[77] Sharma M., Nag M., Del Prete G.Q. (2024). Minimally Modified HIV-1 Infection of Macaques: Development, Utility, and Limitations of Current Models. Viruses.

[78] Wahl S.M., Redford M., Christensen S., Mack W.J., Cohn J., Janoff E.N., Gange S., Mestecky J., Mofenson L., Kovacs A. (2011). Systemic and mucosal differences in HIV burden, immune, and therapeutic responses. J. Acquir. Immune Defic. Syndr..

[79] Ma Z.M., Stone M., Piatak M., Schweighardt B., Haigwood N.L., Montefiori D., Lifson J.D., Busch M.P., Miller C.J. (2009). High specific infectivity of plasma virus from the pre-ramp-up and ramp-up stages of acute Simian Immunodeficiency Virus infection. J. Virol..

[80] Vaidya N.K., Ribeiro R.M., Miller C.J., Perelson A.S. (2010). Viral dynamics during primary Simian Immunodeficiency Virus infection: Effect of time-dependent virus infectivity. J. Virol..

[81] Simonetti F.R., Sobolewski M.D., Fyne E., Shao W., Spindler J., Hattori J., Anderson E.M., Watters S.A., Hill S., Wu X. (2016). Clonally expanded CD4+ T cells can produce infectious HIV-1 in vivo. Proc. Natl. Acad. Sci. USA.

[82] Coffin J.M., Hughes S.H. (2021). Clonal expansion of infected CD4+ T cells in people living with HIV. Viruses.

[83] Veenhuis R.T., Kwaa A.K., Garliss C.C., Latanich R., Salgado M., Pohlmeyer C.W., Nobles C.L., Gregg J., Scully E.P., Bailey J.R. (2018). Long-term remission despite clonal expansion of replication-competent HIV-1 isolates. JCI Insight.

[84] Kelley C.F., Kitchen C.M., Hunt P.W., Rodriguez B., Hecht F.M., Kitahata M., Crane H.M., Willig J., Mugavero M., Saag M. (2009). Incomplete peripheral CD4+ cell count restoration in HIV-infected patients receiving long-term antiretroviral treatment. Clin. Infect. Dis..

[85] Battegay M., Nüesch R., Hirschel B., Kaufmann G.R. (2006). Immunological recovery and antiretroviral therapy in HIV-1 infection. Lancet Infect. Dis..

[86] Sandberg J.K., Fast N.M., Jordan K.A., Furlan S.N., Barbour J.D., Fennelly G., Dobroszycki J., Spiegel H.M., Wiznia A., Rosenberg M.G. (2003). HIV-specific CD8+ T cell function in children with vertically acquired HIV-1 infection is critically influenced by age and the state of the CD4+ T cell compartment. J. Immunol..

[87] Markowitz M., Louie M., Hurley A., Sun E., Di Mascio M., Perelson A.S., Ho D.D. (2003). A Novel Antiviral Intervention Results in More Accurate Assessment of Human Immunodeficiency Virus Type 1 Replication Dynamics and T-Cell Decay In Vivo. J. Virol..

[88] Marinda E., Humphrey J.H., Iliff P.J., Mutasa K., Nathoo K.J., Piwoz E.G., Moulton L.H., Salama P., Ward B.J., Group Z.S. (2007). Child mortality according to maternal and infant HIV status in Zimbabwe. Pediatr. Infect. Dis. J..

[89] Ferrand R.A., Corbett E.L., Wood R., Hargrove J., Ndhlovu C.E., Cowan F.M., Gouws E., Williams B.G. (2009). AIDS among older children and adolescents in Southern Africa: Projecting the time course and magnitude of the epidemic. AIDS.

[90] Shearer W.T., Rosenblatt H.M., Gelman R.S., Oyomopito R., Plaeger S., Stiehm E.R., Wara D.W., Douglas S.D., Luzuriaga K., McFarland E.J. (2003). Lymphocyte subsets in healthy children from birth through 18 years of age: The Pediatric AIDS Clinical Trials Group P1009 study. J. Allergy Clin. Immunol..

[91] Schatorjé E.J., Gemen E.F., Driessen G.J., LeuveniNK J., van Hout R.W., de Vries E. (2012). Paediatric reference values for the peripheral T cell compartment. Scand. J. Immunol..

[92] Passaes C., Desjardins D., Chapel A., Monceaux V., Lemaitre J., Mélard A., Perdomo-Celis F., Planchais C., Gourvès M., Dimant N. (2024). Early antiretroviral therapy favors post-treatment SIV control associated with the expansion of enhanced memory CD8+ T-cells. Nat. Commun..

[93] McBrien J.B., Kumar N.A., Silvestri G. (2018). Mechanisms of CD8+ T cell-mediated suppression of HIV/SIV replication. Eur. J. Immunol..

[94] Klatt N.R., Shudo E., Ortiz A.M., Engram J.C., Paiardini M., Lawson B., Miller M.D., Else J., Pandrea I., Estes J.D. (2010). CD8+ lymphocytes control viral replication in SIVmac239-infected rhesus macaques without decreasing the lifespan of productively infected cells. PLoS Pathog..

[95] Wong J.K., Strain M.C., Porrata R., Reay E., Sankaran-Walters S., Ignacio C.C., Russell T., Pillai S.K., Looney D.J., Dandekar S. (2010). In vivo CD8+ T-cell suppression of siv viremia is not mediated by CTL clearance of productively infected cells. PLoS Pathog..

[96] Elemans M., Seich al Basatena N.K., Klatt N.R., Gkekas C., Silvestri G., Asquith B. (2011). Why don’t CD8+ T cells reduce the lifespan of SIV-infected cells in vivo?. PLoS Comput. Biol..

[97] Seich al Basatena N.K., Chatzimichalis K., Graw F., Frost S.D., Regoes R.R., Asquith B. (2013). Can non-lytic CD8+ T cells drive HIV-1 escape?. PLoS Pathog..

[98] Spits H.B., Mudrikova T., Schellens I.M., Wensing A.M., Prins J.M., Feuth T., Spierings E., Nijhuis M., van Baarle D., Borghans J.A. (2016). The presence of protective cytotoxic T lymphocytes does not correlate with shorter lifespans of productively infected cells in HIV-1 infection. AIDS.

[99] Balamurali M., Petravic J., Loh L., Alcantara S., Kent S.J., Davenport M.P. (2010). Does cytolysis by CD8+ T cells drive immune escape in HIV infection?. J. Immunol..

[100] Wick W.D., Yang O.O. (2012). Biologically-directed modeling reflects cytolytic clearance of SIV-infected cells in vivo in macaques. PLoS ONE.

[101] Gadhamsetty S., Coorens T., de Boer R.J. (2016). Notwithstanding circumstantial alibis, cytotoxic T cells can be major killers of HIV-1-infected cells. J. Virol..

[102] Zanoni M., Palesch D., Pinacchio C., Statzu M., Tharp G.K., Paiardini M., Chahroudi A., Bosinger S.E., Yoon J., Cox B. (2020). Innate, non-cytolytic CD8+ T cell-mediated suppression of HIV replication by MHC-independent inhibition of virus transcription. PLoS Pathog..

[103] Alter G., Altfeld M. (2009). NK cells in HIV-1 infection: Evidence for their role in the control of HIV-1 infection. J. Intern. Med..

[104] O’Connor G.M., Holmes A., Mulcahy F., Gardiner C.M. (2007). Natural Killer cells from long-term non-progressor HIV patients are characterized by altered phenotype and function. Clin. Immunol..

[105] Martin M.P., Qi Y., Gao X., Yamada E., Martin J.N., Pereyra F., Colombo S., Brown E.E., Shupert W.L., Phair J. (2007). Innate partnership of HLA-B and KIR3DL1 subtypes against HIV-1. Nat. Genet..

[106] Bostik P., Takahashi Y., Mayne A.E., Ansari A.A. (2010). Innate immune natural killer cells and their role in HIV and SIV infection. HIV Ther..

[107] Reeves R.K., Li H., Jost S., Blass E., Li H., Schafer J.L., Varner V., Manickam C., Eslamizar L., Altfeld M. (2015). Antigen-specific NK cell memory in rhesus macaques. Nat. Immunol..

[108] Mikulak J., Oriolo F., Zaghi E., Di Vito C., Mavilio D. (2017). Natural killer cells in HIV-1 infection and therapy. AIDS.

[109] Parato K.G., Kumar A., Badley A.D., Sanchez-Dardon J.L., Chambers K.A., Young C.D., Lim W.T., Kravcik S., Cameron D.W., Angel J.B. (2002). Normalization of natural killer cell function and phenotype with effective anti-HIV therapy and the role of IL-10. AIDS.

[110] Alter G., Malenfant J.M., Delabre R.M., Burgett N.C., Yu X.G., Lichterfeld M., Zaunders J., Altfeld M. (2004). Increased natural killer cell activity in viremic HIV-1 infection. J. Immunol..

[111] Kottilil S., Shin K., Planta M., McLaughlin M., Hallahan C., Ghany M., Chun T.W., Sneller M., Fauci E.A. (2004). Expression of chemokine and inhibitory receptors on natural killer cells: Effect of immune activation and HIV viremia. J. Infect. Dis..

[112] Mavilio D., Lombardo G., Benjamin J., Kim D., Follman D., Marcenaro E., O’Shea M.A., Kinter A., Kovacs C., Moretta A. (2005). Characterization of CD56–/CD16+ natural killer (NK) cells: A highly dysfunctional NK subset expanded in HIV-infected viremic individuals. Proc. Natl. Acad. Sci. USA.

[113] Azzoni L., Papasavvas E., Chehimi J., Kostman J.R., Mounzer K., Ondercin J., Perussia B., Montaner L.J. (2002). Sustained impairment of IFN-*γ* secretion in suppressed HIV-infected patients despite mature NK cell recovery: Evidence for a defective reconstitution of innate immunity. J. Immunol..

[114] Zaghouani H., Hoeman C.M., Adkins B. (2009). Neonatal immunity: Faulty T-helpers and the shortcomings of dendritic cells. Trends Immunol..

[115] Soghoian D.Z., Jessen H., Flanders M., Sierra-Davidson K., Cutler S., Pertel T., Ranasinghe S., Lindqvist M., Davis I., Lane K. (2012). HIV-specific cytolytic CD4 T cell responses during acute HIV infection predict disease outcome. Sci. Transl. Med..

[116] Soghoian D.Z., Streeck H. (2010). Cytolytic CD4+ T cells in viral immunity. Expert Rev. Vaccines.

[117] Wegmann T.G., Lin H., Guilbert L., Mosmann T.R. (1993). Bidirectional cytokine interactions in the maternal-fetal relationship: Is successful pregnancy a TH2 phenomenon?. Immunol. Today.

[118] Pazos M., Sperling R.S., Moran T.M., Kraus T.A. (2012). The influence of pregnancy on systemic immunity. Immunol. Res..

[119] Sperling R.S., Engel S.M., Wallenstein S., Kraus T.A., Garrido J., Singh T., Kellerman L., Moran T.M. (2012). Immunogenicity of trivalent inactivated influenza vaccination received during pregnancy or postpartum. Obstet. Gynecol..

