# Supplementary material for: Comparative Analysis of Within-Host Dynamics of Acute Infection and Viral Rebound Dynamics in Postnatally SHIV-Infected ART-Treated Infant Rhesus Macaques
Source: Viruses. 2026 Sep 29;18(10):1076. doi: 10.3390/v18101076 (PMC13709779; doi:10.3390/v18101076)
Supplement: Supplementary file 1 [file viruses-18-01076-s001.zip › B2AcRe Supplementary Figures.pdf]

Comparative analysis of within-host dynamics of acute infection and rebound dynamics in postnatally SHIV-infected ART-treated infant rhesus macaques

Ellie Mainou[1], Stella J Berendam[2], Veronica Obregon-Perko[3], Emilie A Uffman[4], Caroline T Phan[4], George M Shaw[5], Katherine J Bar[5], Mithra R Kumar[6], Emily J Fray[7], Janet M Siliciano[8], Robert F Siliciano[8], Guido Silvestri[9], Sallie R Permar[10], Genevieve G Fouda[10], Janice McCarthy[11], Ann Chahroudi[3], Jessica M Conway[12+], Cliburn Chan[11+]

[1] Department of Biology, Pennsylvania State University, University Park, PA, USA.
[2] GlaxoKlineSmith, Rockville, MD, USA.
[3] Department of Pediatrics, Emory University School of Medicine, Atlanta, GA 30322, USA.
[4] Duke Human Vaccine Institute, Duke University Medical Center, Durham, NC, USA.
[5] Perelman School of Medicine, University of Pennsylvania, Philadelphia, Pennsylvania, USA.
[6] Department of Pharmacology and Molecular Sciences, Johns Hopkins University School of Medicine, Baltimore, MD, USA.
[7] Department of Biochemistry and Molecular Biology, Johns Hopkins University School of Medicine, Baltimore, MD, USA.
[8] Department of Medicine, Johns Hopkins University School of Medicine, Baltimore, MD, USA.
[9] Yerkes National Primate Research Center, Emory University, Atlanta, Georgia, USA.
[10] Department of Pediatrics, Weill Cornell Medicine, New York, NY, USA.
[11] Department of Biostatistics and Bioinformatics, Duke University Medical Center, Durham, NC, USA.
[12]Department of Mathematics, Pennsylvania State University, University Park, PA, USA.
[+]These authors have contributed equally. \\

Supplementary Information

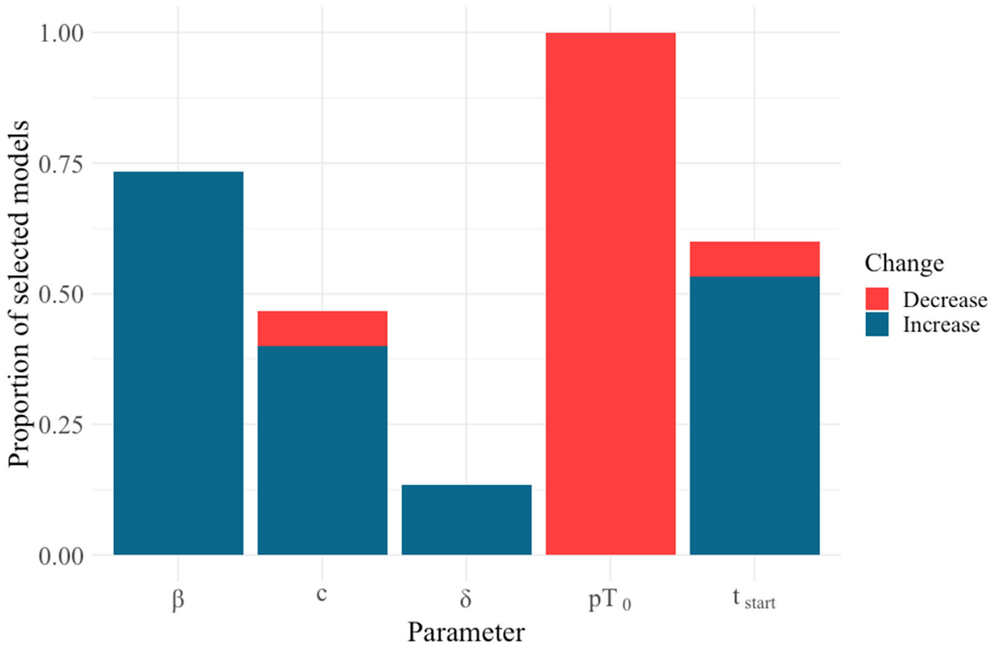

Supplementary Figure S1: Bar chart depicting the proportion times of a parameter is predicted to differ between acute infection and viral rebound in all selected models with ΔAIC<8 and *S*<80. A total of 15 models were selected. Viral clearance rate (c) is predicted to be different between acute infection and viral rebound in 7 models; death rate of infected cells (δ) in 2 models; start of exponential viral growth (t_{start}) in 9 models; mass-action infectivity (β) in 11 models and the product of viral production rate (p) and start of initial number of target cells (T_0) in all 15 models. Red slices signify a decrease in the value of the parameter for rebound, whereas blue-toned slices depict an increase in the rebound value of the parameter. Allowing for an increased *S* threshold introduces variability in the predicted association between the stage of the infection and the mean value of the viral clearance rate and the onset of viral exponential growth.

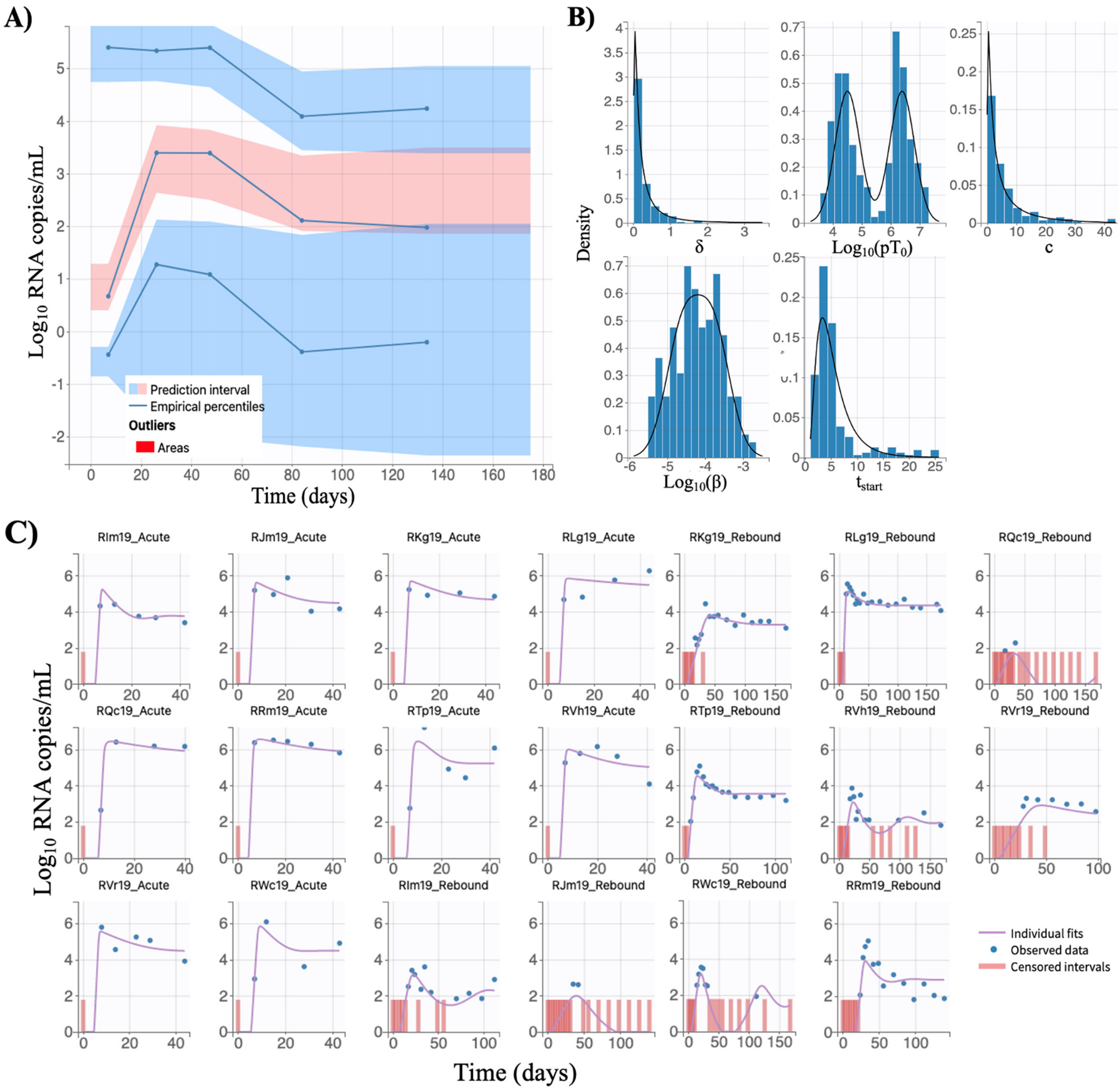

Supplementary Figure S2: Example of one of the selected models selected. In this model we fix the median viral clearance rate to c=3 day^{-1}for acute infection, and we assume that the product of viral production rate and initial number of target cells (pT_0) as well as the mass action infectivity (β) are difference between acute and rebound and add a correlation between c and β. A) Visual predictive check estimated from the model. B) Empirical distribution of estimated individual parameter values (blue bars) along with theoretical distribution (black line). C) Viral

load measurements for each RM (points) along with the fitted curve (line). Measurements below the limit of detection are depicted in red bars.

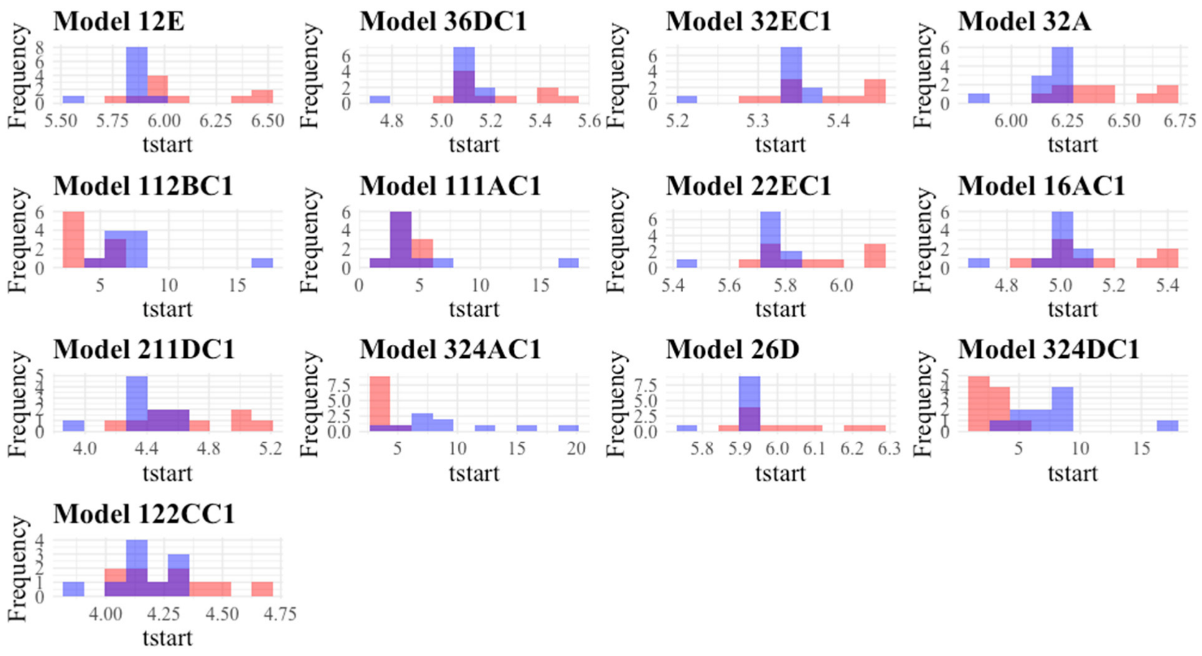

Supplementary Figure S3: Distribution of the onset of exponential viral growth (t_{start}) for acute infection (red) and viral rebound (blue) for all selected models.

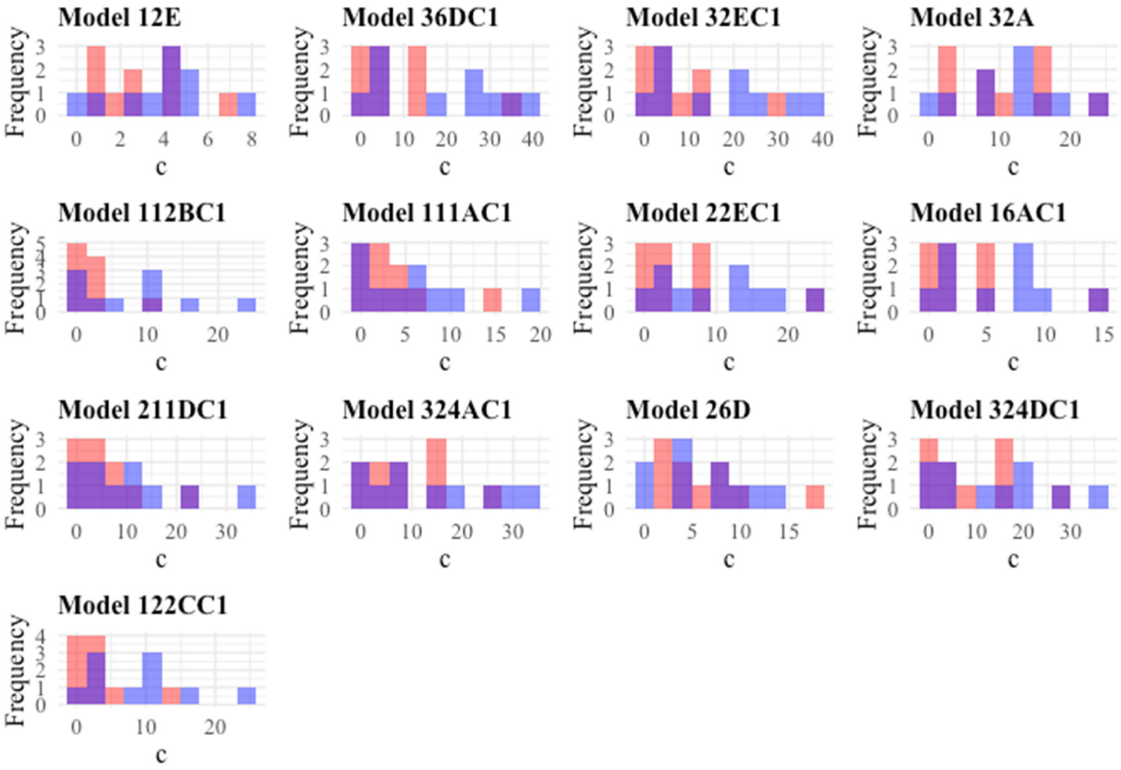

Supplementary Figure S4: Distribution of the estimated viral clearance rate (c) for acute infection (red) and viral rebound (blue) for all selected models.

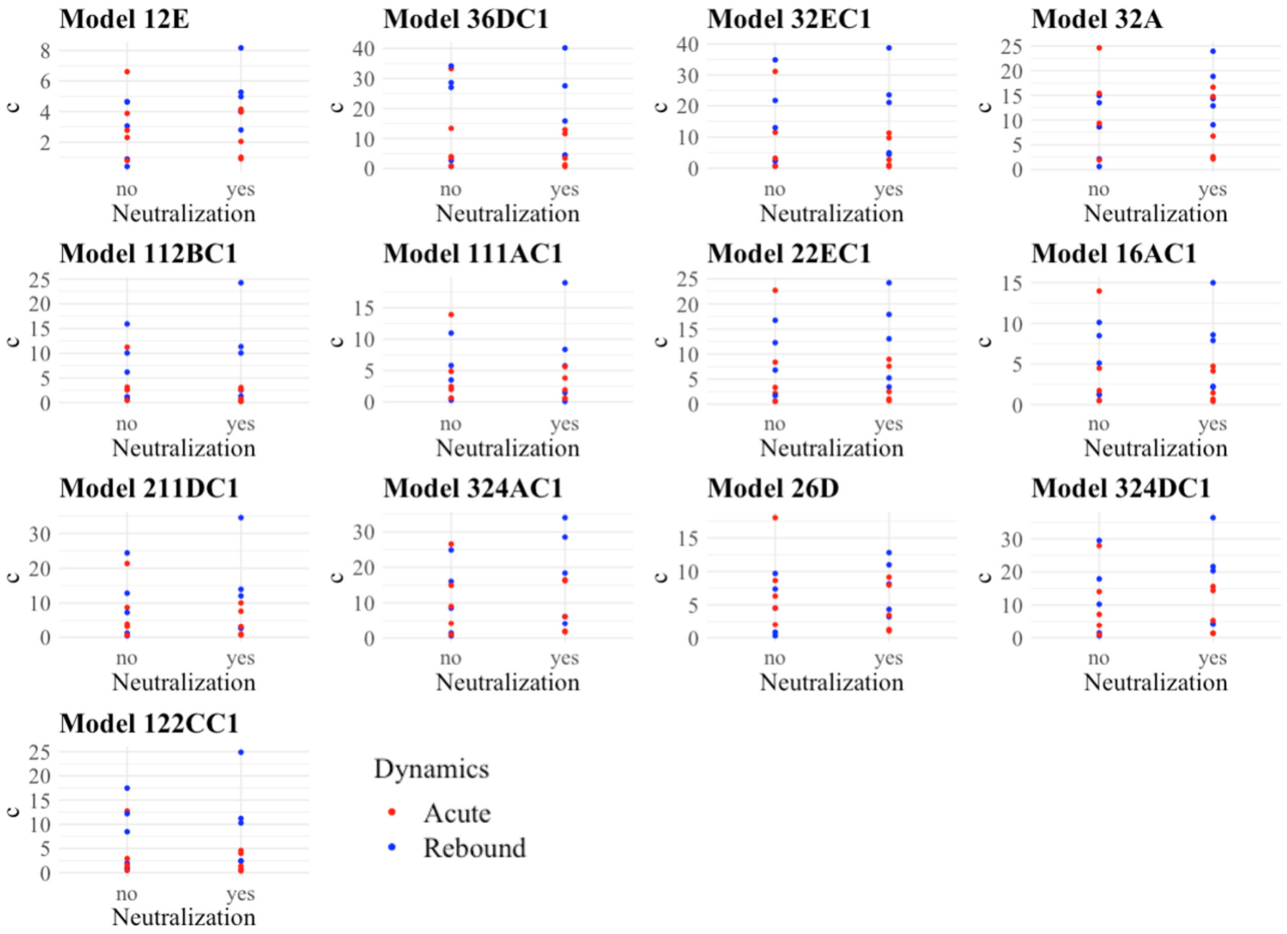

Supplementary Figure S5: Scatterplot of the estimated viral clearance rate per day (c) for acute infection (red) and viral rebound (blue) for all selected models based on whether the RM developed antibody neutralizing responses.

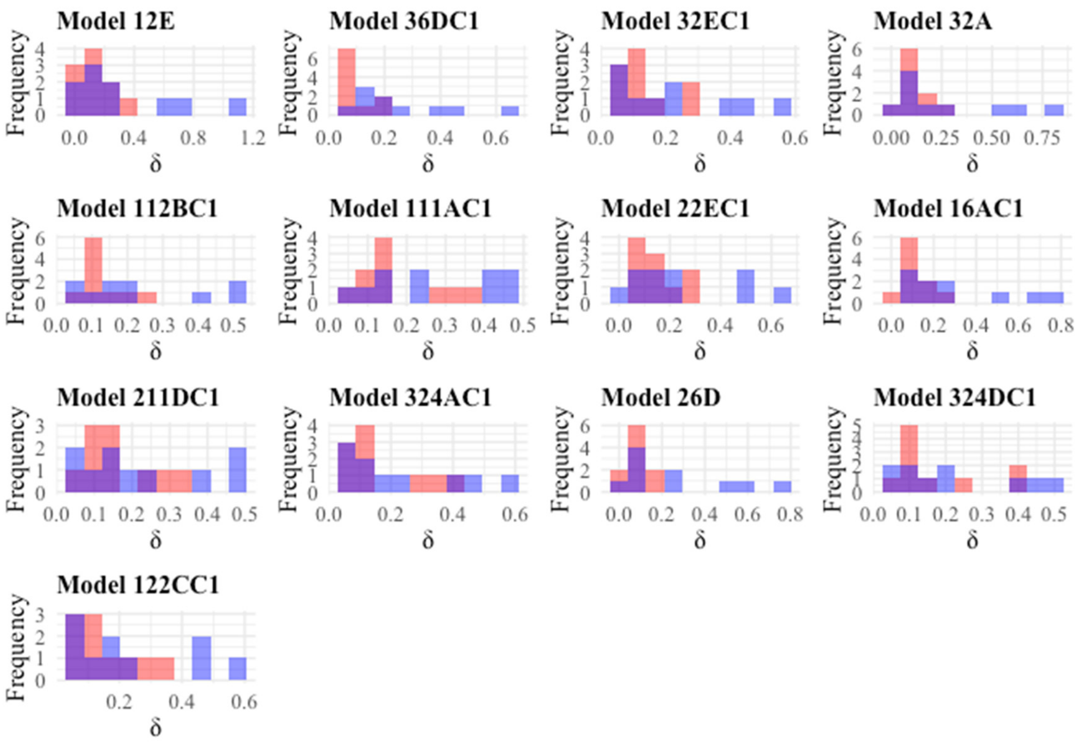

Supplementary Figure S6: Distribution of the estimated infected cell death rate (δ) for acute infection (red) and viral rebound (blue) for all selected models.

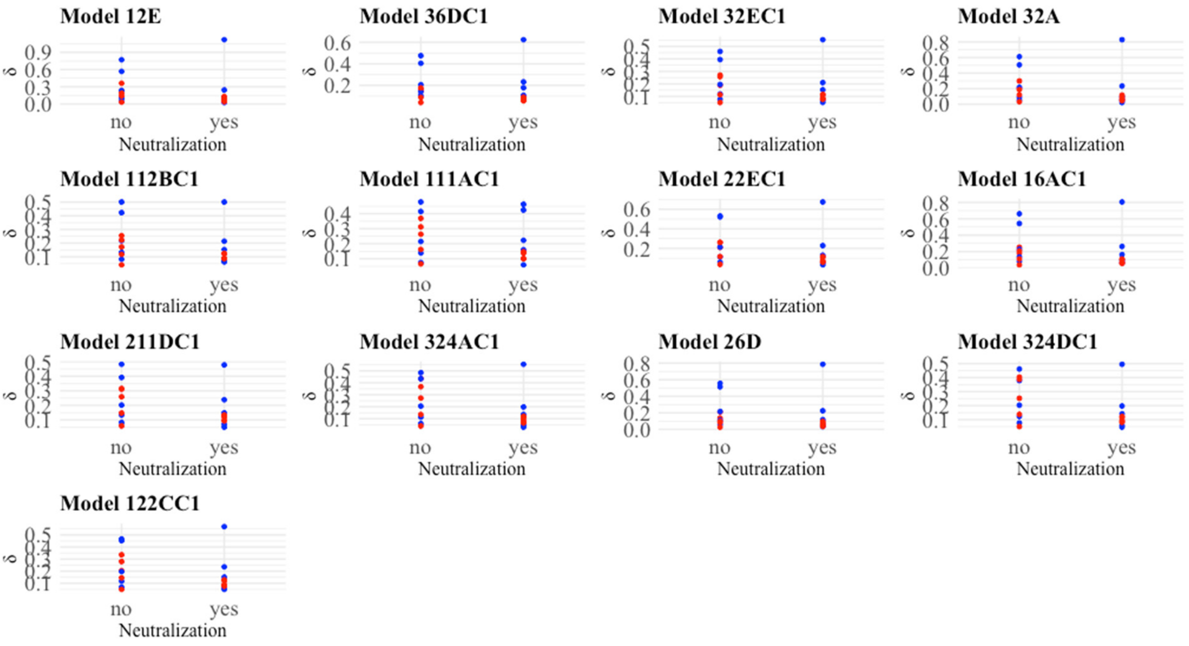

Supplementary Figure S7: Scatterplot of the estimated death rate of infected cells per day (δ) for acute infection (red) and viral rebound (blue) for all selected models based on whether the RM developed antibody neutralizing responses.
